# Editing the Embryonic Brain: How In Utero Electroporation Reveals the Secrets of Cortical Development

**DOI:** 10.3390/ijms27219760

**Published:** 2026-10-30

**Authors:** Ermoshina Anastasiia, Okhalnikov Alexandr, Kolbasov Pavel, Dubrovina Assol’, Tarabykin Victor, Gavrish Maria

**Affiliations:** 1 Institute of Neuroscience, Lobachevsky State University of Nizhny Novgorod, 23 Gagarin Ave., 603022 Nizhny Novgorod, Russia; 2 Department of Genetics and Life Sciences, Sirius University of Science and Technology, 1 Olympic Ave, 354340 Sirius Federal Territory, Russia; 3 Institute of Theoretical and Experimental Biophysics of Russian Academy of Sciences, 142290 Pushchino, Russia

**Keywords:** corticogenesis, cell cycle, thymidine analogues, axonal navigation

## Abstract

The development of the mammalian cerebral cortex follows a tightly regulated dynamic molecular program, the critical stages and key molecular players of which remain to be fully elucidated. Detailed visualization of these processes, along with functional genomic studies, will significantly advance our understanding of neurogenesis and its associated human disorders. Such developmental aberrations often entail irreversible changes that lead to congenital malformations of varying severity. *In utero* electroporation (IUE) has emerged as a widely utilized *in vivo* transfection and imaging technique. When coupled with advanced technologies such as genome editing and systems for spatiotemporal and tissue-specific gene expression, IUE offers unprecedented opportunities to dissect the molecular mechanisms underlying embryonic cortical development both under normal conditions and in various pathological processes. This review highlights the technical utility of IUE and provides examples of experimental designs used to investigate stages of neurogenesis. Particular attention has been paid to studying the cell cycle using thymidine analogues, the differentiation of neuronal progenitor cells, and axonal guidance.

## 1. Introduction

The development of the cerebral cortex proceeds through sequential, tightly organized molecular and cellular events. The proper assembly of this complex structure depends on the precise coordination of all components within the system [1,2,3]. Disruptions occurring at any of the developmental stages result in congenital malformations of varying severity [4]. Consequently, investigating the molecular and cellular aspects of neocortical development (corticogenesis) is critical to elucidating the pathogenic mechanisms of these congenital disorders, identifying preventive measures, and fostering the development of therapeutic intervention strategies.

A variety of *in vitro* models are currently utilised to study corticogenesis, including embryonic stem cells (ESCs) and induced pluripotent stem cells (iPSCs) [5]—as well as primary mixed neuron–glial cultures [6].

However, the development of a structure as complex as the cerebral cortex is governed by an intricate array of microenvironmental and systemic factors that cannot be fully recapitulated in *in vitro* systems or captured through *post mortem* analyses [7]. Therefore, it must be investigated *in vivo* using laboratory animal models, spanning from the early stages of embryonic development to full maturation Within this framework, methodological approaches that enable precise manipulation of the model organism are of particular interest. These include the generation of transgenic animal lines (Cre/loxP, etc.), which enable tissue- and cell-specific gene modification and, when inducible systems (CreER) are used, temporally controlled modification as well [8,9,10]. Additionally, the application of recombinant viral vectors—adeno-associated viruses (AAVs) and lentiviruses (LVs)—to these models represents a more flexible method, providing spatially and temporally controlled delivery of genetic constructs to the central nervous system (CNS) [11,12]. Another technique used in *in vivo* studies is ultrasound-guided *in utero* transplantation (IUT) of labeled cells, which enables direct delivery and tracking of cell populations in the developing brain and beyond [13,14].

Nevertheless, these approaches have several limitations. For instance, the generation of transgenic lines carries the risk of insertional mutagenesis, wherein the exogenous gene may disrupt endogenous genes, alter regulatory elements, or modify chromatin structure, potentially leading to confounding phenotypic consequences and skewed experimental outcomes [15,16]. Moreover, because transgenic modifications are heritable, they are not ideal for modeling certain human neurological disorders driven by de novo (spontaneous) mutations, which instead require somatic gene delivery directly into the native, developing brain [17,18]. Alternatively, while viral delivery systems allow for such somatic approaches, their primary drawbacks include the risk of triggering an inflammatory or immune response [19,20] as well as the potential for epigenetic silencing of the viral transgene by host defense mechanisms [21].

A major breakthrough in developmental neurobiology occurred in the early 2000s, when two independent research groups developed the *in utero* electroporation technique, effectively overcoming these historical limitations [22,23].

IUE has since become widely used in developmental neurobiology because it enables visualization of diverse cellular processes during corticogenesis. For example, it allows monitoring of neuronal migration in organotypic brain slices, which maintain *in vivo*-like development for at least 72 h [24,25]. Delivery of specific genetic constructs further permits real-time assessment of how altered target-gene expression affects migration dynamics by live-cell confocal imaging [25,26]. When combined with cell-type-specific reporter plasmids, multiplexed immunohistochemistry, high-resolution confocal microscopy, and spatial cell-distribution analysis, IUE enables investigation of gene function in defined brain regions and neuronal lineages, fate-mapping of transfected cells, and dissection of molecular-genetic pathways of neurogenesis under near-physiological conditions [27,28,29].

Beyond the study of normal development, IUE is also employed to model brain pathologies. For instance, expression of an active form of *RHEB* (Ras homolog enriched in brain) enabled the generation of a model of focal cortical dysplasia: spontaneous seizures were observed in 8 out of 11 mice, with an average of 5.7 seizures per day [30]. In another study on lissencephaly, suppression of *CEP85L* (Centrosomal protein of 85 kDa-like) expression via IUE led to impaired neuronal migration [31]. Furthermore, IUE is used to model genetic disorders of corticogenesis. For example, knockdown of the *MTSS2* (MTSS I-BAR domain containing 2) gene, associated with microcephaly, using shRNA delivered by IUE resulted in impaired radial glial cell division and neuronal progenitor migration [32]. Thus, IUE enables investigation of the relationship between specific genetic perturbations and pathological changes in brain development.

Although IUE has become a widely used tool for studying corticogenesis, it is not without limitations, and its suitability depends on the specific experimental objectives. Table 1 provides a comparative overview of IUE and other commonly used *in vivo* approaches, including transgenic models, viral delivery systems, and ultrasound-guided *in utero* transplantation (IUT), highlighting their respective targeting specificity, efficiency, duration of gene expression, technical complexity, and main experimental limitations.

The fundamental principles of IUE and its use in studying key aspects of cortical development, particularly radial migration and cell-fate specification, have been extensively covered in previous reviews [55,64,65]. 

In this review, we aim to focus on several crucial yet frequently overlooked applications of the IUE methodology, namely, the investigation of cell cycle dynamics, neural progenitor cell differentiation, and the axonal guidance of newborn neurons. We provide a comprehensive overview spanning from experimental design and execution to the subsequent analysis of the acquired data. We will also show examples of such experiments from our laboratory.

## 2. Principle of the Method and Main Approaches

Electroporation relies on the capacity of negatively charged DNA molecules—most commonly plasmid vectors—to penetrate the cell through transient pores formed in the plasma membrane upon exposure to an electric field [66]. The technique was first employed in 1982 by T. Wong and E. Neumann for the *in vitro* delivery of plasmid DNA into LM (TK-) cells, a line derived from mouse fibroblasts [67]. Subsequently, the method underwent a series of modifications. For instance, several studies demonstrated the feasibility of using electroporation to deliver plasmid vectors into the organs of adult rodents *in vivo* [68,69], while the earliest experiments in avian models were performed in ovo on chick embryos [70]. The first reports of *in utero* electroporation in the scientific community are attributed to two independent research groups [22,23]; in addition, several other studies published in 2001 described the adaptation of conventional electroporation protocols for IUE applications [22,71].

**Figure 1:**
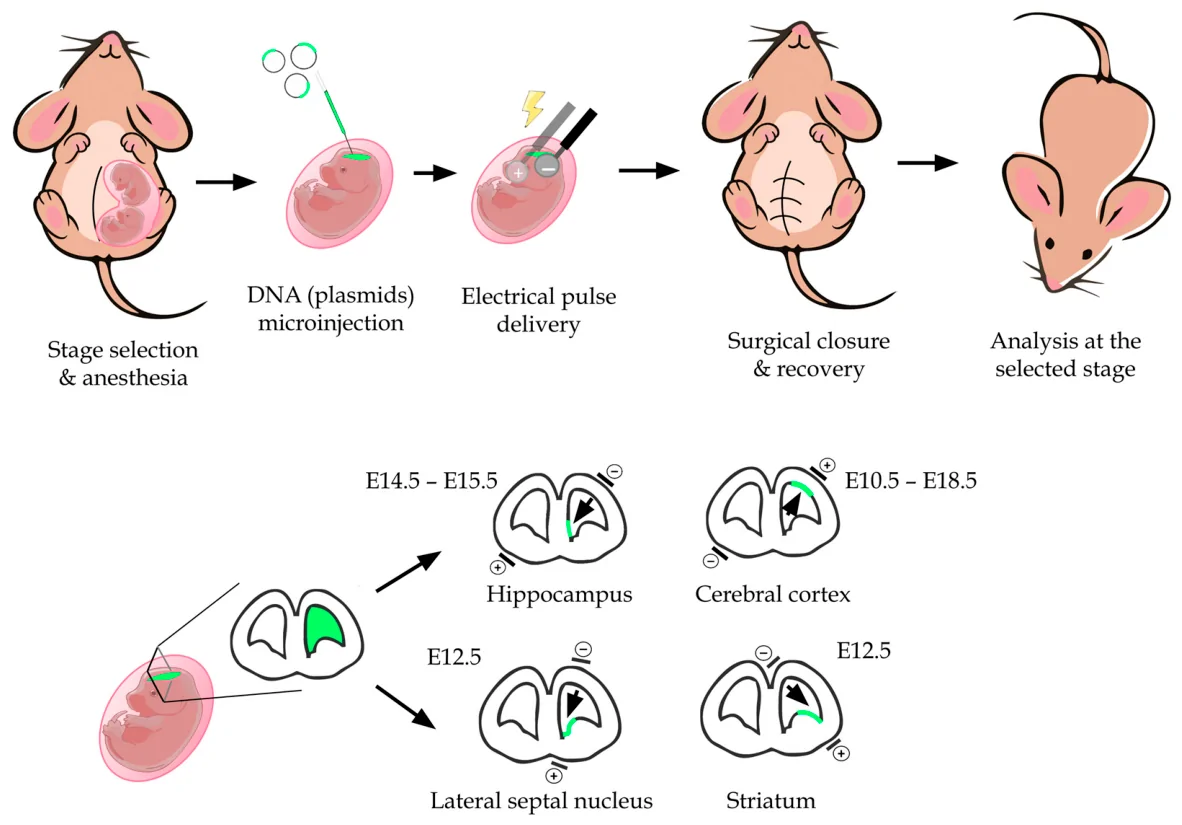
The schematic illustration of the IUE procedure and the selected area. The area highlighted in green indicates the site where the genetic construct was introduced. Created in Inkscape 1.4.2 (The Inkscape Team, Boston, MA, USA).

As a technique, IUE shares a set of common methodological features that have been detailed in a number of protocol papers (Figure 1) [66,72,73,74,75].

**Figure 2:**
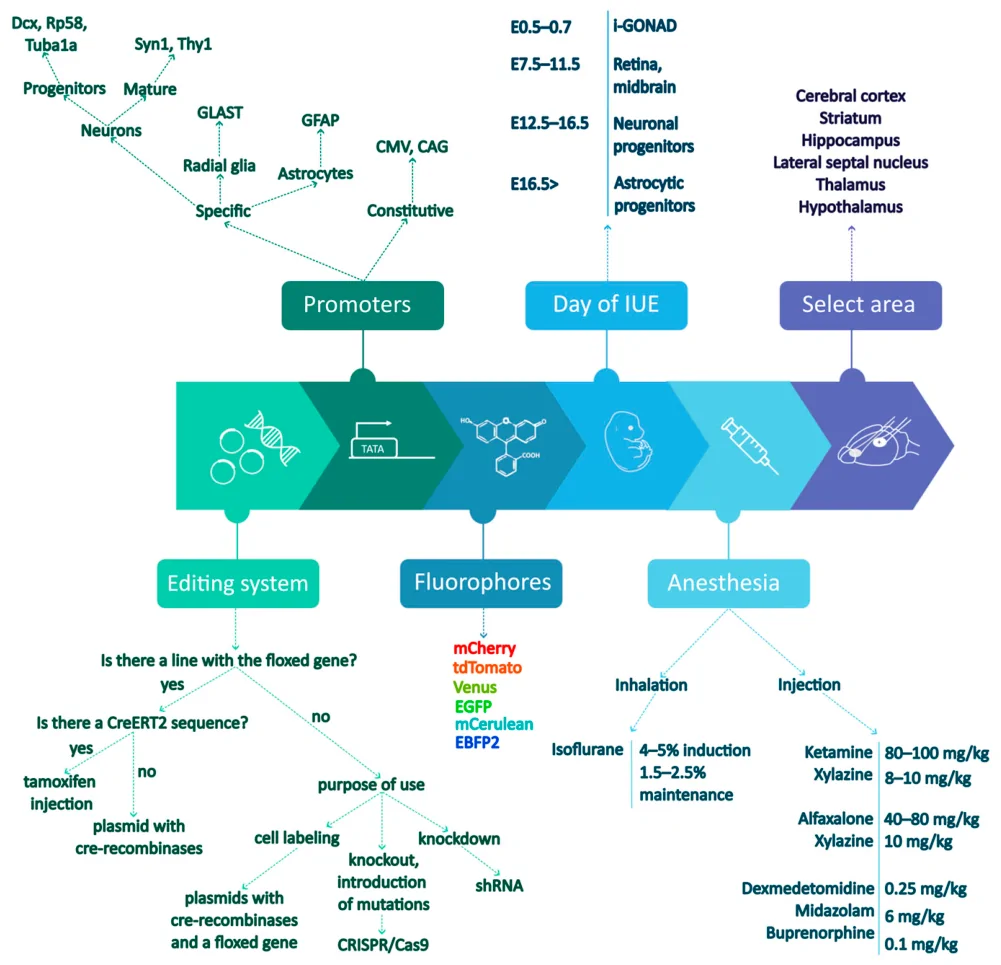
Selection of key electroporation parameters according to the aims of the study. Dcx—doublecortin, Rp58—Repressor Protein 58, Tuba1a—Tubulin Alpha 1a, Syn1—Synapsin 1, Thy1—Thymocyte antigen 1, GLAST—Glutamate–aspartate transporter, GFAP—Glial fibrillary acidic protein, CMV—Cytomegalovirus, CAG—CMV early enhancer/chicken β-actin, E—embryonic day, CreERT2—Tamoxifen-inducible Cre recombinase (second generation), shRNA—Small hairpin ribonucleic acid, EGFP—Enhanced Green Fluorescent Protein, and EBFP2—Enhanced Blue Fluorescent Protein 2. Created in Inkscape 1.4.2 (The Inkscape Team, Boston, MA, USA).

Nevertheless, many experimental parameters may be adjusted according to the specific aims and objectives of a given study (Figure 2), as discussed in greater detail below.

### 2.1. Editing Systems Used for IUE

The gene-editing strategy is selected according to the specific aims of the study and the genetic tools available. Thus, knockout of a target gene or the introduction of a specific mutation is typically achieved using the CRISPR/Cas9 system, whereas knockdown is accomplished via RNA interference using shRNA [76,77,78].

**Figure 3:**
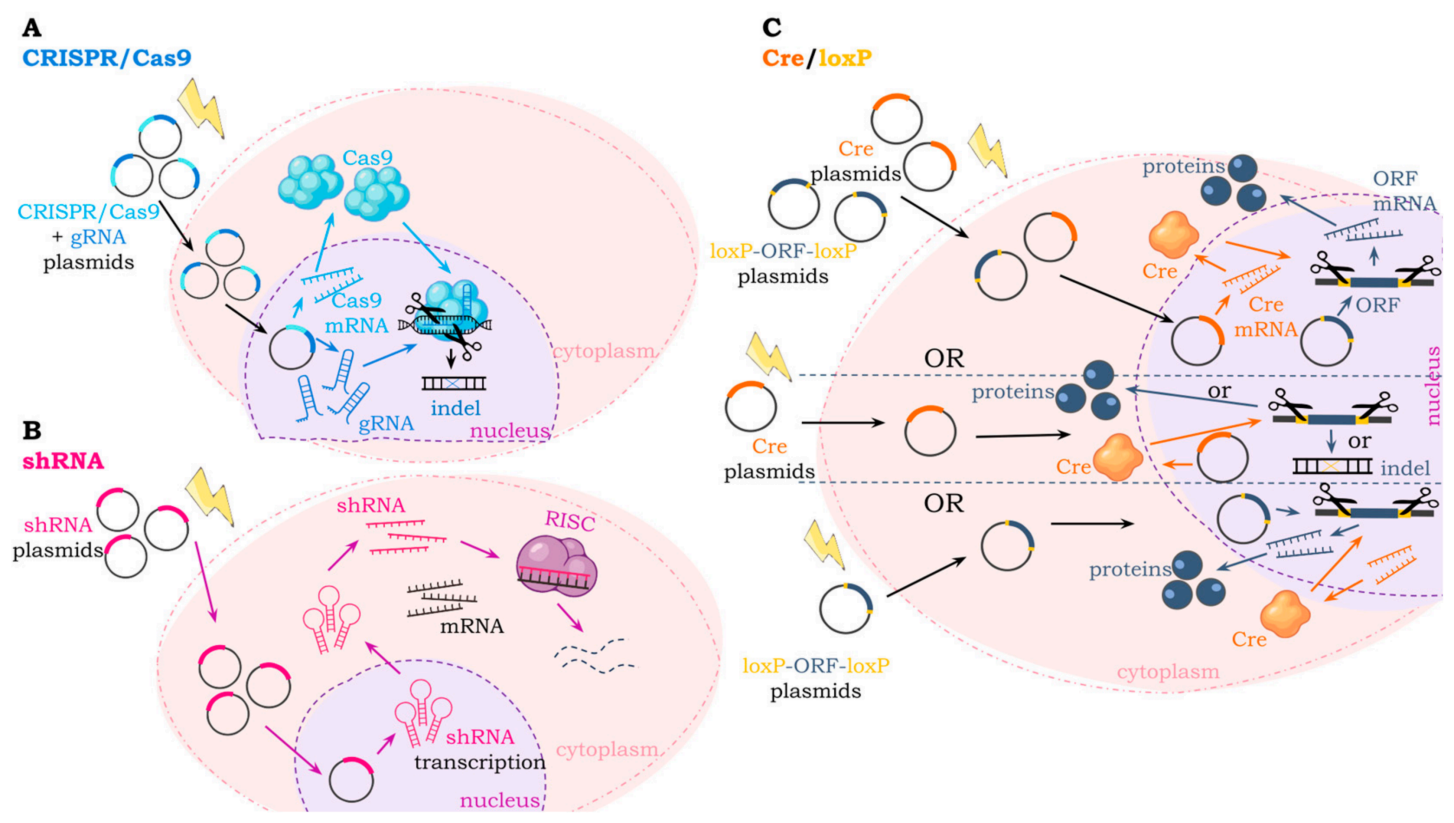
The main genetic manipulation strategies used in IUE. (**A**)—CRISPR/Cas9, (**B**)—shRNA, (**C**)—Cre/LoxP. shRNA—Small hairpin ribonucleic acid, mRNA—messenger ribonucleic acid, ORF—open reading frame, and RISC—RNA-induced silencing complex. Created in Inkscape 1.4.2 (The Inkscape Team, Boston, MA, USA).

In CRISPR/Cas9, a construct encoding Cas9 and a guide RNA (gRNA) is delivered into the cell. The gRNA binds the complementary target sequence in complex with the Cas9 nuclease, which induces a double-strand DNA break. This break is repaired by either non-homologous end joining (NHEJ) or homology-directed repair (HDR) (Figure 3A).

RNA interference is achieved through electroporation of an shRNA-encoding plasmid or synthetic RNA. Subsequently, the RNA undergoes maturation in the cytoplasm, as a result of which it forms a complex with RISC (RNA-induced silencing complex) and becomes capable of binding and degrading target RNAs (Figure 3B) [65].

The i-GONAD (Improved Genome-editing via Oviductal Nucleic Acids Delivery) method merits separate consideration. Its distinctive feature is that, during surgery at E0.7, the ovaries and oviducts are exposed through a dorsolateral incision (in the original GONAD protocol, surgery was performed at E1.5 [79]), and genome-editing reagents are introduced into the lumen of the oviduct using a glass capillary pipette. Immediately after injection of the solution, the entire oviduct is subjected to electroporation using tweezer-type electrodes [80], thereby making the generation of transgenic animals technically simpler by eliminating the step involving the handling of isolated embryos [81]. This approach makes it possible to generate knockouts and introduce mutations, floxed alleles [82], and cassettes of up to 0.9 kb in length.

For the insertion of larger cassettes, the use of Easi-CRISPR (efficient additions with ssDNA inserts–CRISPR) in combination with the more classical zygote microinjection method is recommended [81]. Easi-CRISPR is based on the use of single-stranded DNA (ssDNA) as a donor insert into the edited genome, which is claimed to be a more efficient approach compared with the use of double-stranded DNA (dsDNA) [83]. Another difference from other methods is the introduction of a pre-assembled CRISPR RNP complex, which accelerates the genome-editing process [84]. However, this method is used specifically for genome editing of embryos and the generation of transgenic lines [85].

Easi-CRISPR has also been adapted for the developing brain in an approach termed Breasi-CRISPR (“Brain Easi-CRISPR”). In this case, a pre-formed Cas9/gRNA complex and donor DNA are delivered into cells by IUE. The authors report that endogenous protein can be visualized in individual cells as early as 1 day after electroporation, in up to 50% of electroporated cells and 30% of all cells in the electroporated region. The method is used to tag endogenous proteins of interest with fluorescent sequences, enabling visualization of their dynamics in tissue [86], or to model potentially pathogenic gene variants, such as a *CCND2* gene variant possibly associated with MPPH syndrome (megalencephaly, postaxial polydactyly, polymicrogyria, and hydrocephalus) [87].

Proteins of interest can also be tagged using the SLENDR method (single-cell labeling of endogenous proteins by CRISPR-Cas9-mediated homology-directed repair). In this approach, CRISPR-Cas9 is encoded by the delivered plasmid vector. Insertion of the tag sequence, provided as a single-stranded oligodeoxynucleotide (ssODN), is mediated by homology-directed repair (HDR), which exhibits high accuracy but relatively low efficiency (<10% of the electroporated cells) [88,89]. This subsequently enables researchers to determine the localization and dynamics of endogenous proteins that are difficult to detect by other methods [90].

Unlike the methods described above, which operate at early embryonic stages, the NHEJ mechanism enables DNA integration into both dividing and non-dividing cells, which may be of interest for studies of the brain at postnatal stages. This principle is exploited in the HITI method (homology-independent targeted integration) [91]. In this system, a gRNA target site is designed in the reverse-complementary orientation relative to the genomic target site, so the donor insert is typically integrated in the correct orientation because correct integration disrupts both sites and stabilizes the donor, whereas incorrect integration recreates two gRNA target sites flanking the insert, allowing its excision and reintegration in the correct orientation [91]. This approach has been used, for example, in strategies for editing pathogenic protein variants, in which the insert was a small superexon capable of restoring the expression of functional CFTR (cystic fibrosis transmembrane conductance regulator) protein, which is involved in the pathogenesis of cystic fibrosis [92].

ORANGE (Open Resource for the Application of Neuronal Genome Editing) was developed on the basis of HITI. It is more accurately described as a toolkit comprising a single template vector that can readily be adapted for tagging virtually any target protein as well as a library of ready-to-use knock-in constructs targeting 38 proteins [93].

In contrast to the methods described above, the HiUGE system (Homology-Independent Universal Genome Engineering) is designed for delivery using AAV vectors containing a payload (typically a tag sequence) flanked by a sequence non-homologous to the target gene. This sequence is recognized by a donor-specific gRNA (DS-gRNA), which directs Cas9-mediated autonomous excision and release of the payload, which can then be integrated at the site specified by the gRNA [94]. This method has been used to introduce tags into the sequences of proteins of interest associated with autism [95].

The use of these techniques enables relatively rapid editing; however, it may sometimes be accompanied by off-target effects. For the CRISPR/Cas9 system, this refers to the binding of the Cas9 nuclease–gRNA complex to a non-target sequence that differs from the target sequence by several nucleotides [96,97]. In contrast, off-target effects associated with the use of shRNA are attributed to imperfect matching of shRNA to unintended mRNA targets, which may become more pronounced in the absence of the intended target. Off-target toxicity resulting from disruption of endogenous microRNA processing caused by shRNA overexpression has also been reported [98].

In this section, we have reviewed genome-editing methods and more specialised approaches based on them. However, in some cases, an editing system with greater temporal specificity and dose dependence is required. The Cre-loxP system possesses such properties. In the following section, we will focus on the principles underlying its operation and its potential applications in combination with IUE.

### 2.2. Cre–LoxP-Based Editing Systems Employed in IUE

The Cre-LoxP system comprises the site-specific recombinase Cre, which binds to its target DNA sequence—the loxP sites—and mediates genomic deletion of the sequence between them [99]. These rearrangements are exploited to introduce point mutations [100] to perform knockout [101] and to model the chromosomal translocations [102]. A combination of deletion and inversion events allows switching between expressed sequences [103]. Furthermore, the Cre-LoxP system can be used for the targeted integration of a DNA fragment into a predetermined genomic locus through a process termed recombinase-mediated cassette exchange (Figure 3C) [104,105].

For use in combination with IUE, the most common configuration involves co-delivery of a plasmid encoding Cre recombinase together with a plasmid carrying a floxed gene [106]. Alternatively, mouse lines whose genome harbours a floxed allele may be used [101], or, conversely, mouse lines expressing Cre recombinase may be electroporated with a plasmid carrying the floxed sequence [29]. Cre recombinase can be activated under conditional control. For example, the Cre coding sequence may be driven by a promoter that is active only under defined conditions. This may be a cell-type-specific promoter such as NeuroD, which is active exclusively in postmitotic neuronal precursors [107]. Alternatively, Cre activity can be regulated temporally by administering 4-hydroxytamoxifen (4-OHT) at a chosen time point; in this case, a plasmid encoding a tamoxifen-inducible form of Cre recombinase is electroporated [108].

For clonal lineage tracing during brain development, the MAGIC (multi-address genome-integrating color) Markers labeling system can be employed. It consists of a plasmid carrying four genes encoding fluorescent proteins with distinct spectral properties, flanked by three pairs of incompatible loxP sites, together with a plasmid encoding the self-excising recombinase SeCre. Prior to Cre activity, only the first gene downstream of the promoter is expressed; the recombinase then stochastically recognizes compatible pairs of loxP sites and excises the intervening DNA fragment [109].

Multicolor cell labeling is also the basis of the Brainbow *in vivo* strategy, which employs two genetic recombination mechanisms acting between fluorescent protein-coding cassettes: Cre-mediated excision between pairs of incompatible lox sites arranged such that only a single recombination event is possible, and inversion of DNA segments. Cre activity is induced by tamoxifen administration, although constitutively active Cre may also be used. Delivery of several Brainbow plasmids into a single cell results in the simultaneous expression of multiple fluorescent proteins, thereby generating a broad palette of hues [110,111,112].

Sparse labeling of individual cells can additionally be achieved using the Supernova system. In its IUE-based configuration, Supernova comprises a set of vectors containing TRE-SSR (TRE, tetracycline response element; SSR, site-specific recombinase) and CAG-RT-stop-RT-XFP-tTA (RT, recombinase target site; XFP, fluorescent protein; tTA, tetracycline transactivator). In this system, leaky expression from the TRE drives weak recombinase expression, which excises the RT-stop-RT cassette in a few copies of the CAG-RT-stop-RT-XFP-tTA vector, initiating low-level transcription of XFP and tTA; tTA subsequently binds the TRE, thereby sustaining recombinase expression. The sparseness of labeling can be tuned by adjusting the concentration of the TRE-containing plasmid [113,114].

The Double UP system likewise relies on Cre recombinase. In the absence of Cre, cells carrying the Double UP plasmid express one color (mNeonGreen or mCherry). A Cre-encoding plasmid is delivered into the cells by IUE; where Cre is present, the mNeonGreen or mCherry cassette is excised and replaced at the same locus by mScarlet or GFP. By titrating the amount of Cre, approximately half of the neurons can be labeled with the green or red reporter, serving as internal controls, while the other half carry both the red or green reporter and the experimental manipulation. Consequently, both green and red neurons are distributed throughout the electroporated region. Imaging of a given cortical area therefore permits quantification of green and red cells within the same section, providing the best comparison [25,115]. For example, in the study by English L. et al. (2025), following Cre-mediated excision, mScarlet was expressed together with the sequence of one of the selected proteins, thereby enabling its overexpression [116].

The use of the Cre-loxP system enables tissue-specific and temporally controlled expression [99,117]. However, the potential toxicity caused by off-target effects due to high levels of nuclear Cre should be taken into account [118], as should the possibility of spontaneous recombination [119]. Therefore, to control for these effects in the resulting phenotypes, it is recommended that an additional control expressing Cre but not carrying the floxed gene be used [120]. Thus, the Cre-loxP system has numerous modifications suitable for various experimental tasks; nevertheless, it is not without certain limitations, the significance of which should be assessed by the investigator in each individual case.

### 2.3. Promoters

Owing to advances in molecular technologies, including genome editing, researchers now have access to an extensive repertoire of vectors that permit selective expression of a gene of interest in defined neuronal or astrocytic subpopulations through the appropriate choice of promoter sequence. Tissue-specific promoters, which are active only in particular cell types, are most frequently employed for this purpose, as they restrict undesirable transgene expression, facilitate sustained expression of exogenous DNA, and enhance the overall efficiency of the construct [121,122].

For instance, the GFAP promoter is used to drive transcription in astrocytes, whereas Syn1 or Thy1 are employed for neurons [123]; the doublecortin (Dcx) promoter drives expression in early neuronal cells [124], while expression driven by the repressor protein 58 (Rp58) promoter is largely confined to multipolar neurons [125]. In addition, certain promoters allow gene expression to be initiated at a defined developmental stage. Thus, Tuba1a is expressed only during embryonic stages in differentiating neurons and, transiently, during the early postnatal period [126], whereas Syn1 is active exclusively in mature neurons [127]. The GLAST promoter (glutamate–aspartate transporter) is active both in radial glial cells (RGCs) and in many astrocytes of the developing central nervous system (CNS), but its activity is downregulated in most astrocytes at postnatal stages in mice. In the mature CNS, the highest GLAST promoter activity has been observed in radial glia, such as those localized in the subgranular/border layer of the hippocampal dentate gyrus [128]. The Nestin promoter can be used to modulate gene expression levels across the entire neural stem/progenitor cell (NSC) population [74,129].

Constitutive promoters such as CMV and CAG (CMV early enhancer/chicken β-actin) are also available [130]; these are not cell-type-specific and maintain moderate expression levels into the postnatal period. At present, however, the CAG promoter is generally preferred, as its efficiency in IUE experiments has been confirmed empirically [131], whereas CMV-driven expression is less stable over time owing to epigenetic silencing (in cell culture studies, expression declines markedly as early as 7 days after transfection) and depends on the differentiation state of the cells [132,133,134].

Finally, the promoter must be compatible with the type of transcript to be generated. For example, mRNA synthesis requires an RNA polymerase II promoter, whereas shRNA production requires an RNA polymerase III promoter. In the former case, the strong constitutive CMV and CAG promoters are most commonly used, whereas the human U6 and H1 promoters are employed for RNA polymerase III [135,136].

### 2.4. Fluorescent Proteins

Electroporated cells in the brain are identified by means of fluorescent proteins encoded within the delivered plasmid vectors [137]. Enhanced green fluorescent protein (EGFP) remains the gold standard and is widely used in the field [138,139]. Its cytotoxicity at high expression levels should nevertheless be taken into account [140,141,142]. Numerous modified variants and analogues of EGFP with improved properties are currently available. For example, enhanced yellow fluorescent protein (EYFP)—an EGFP variant with a red-shifted spectrum—is widely used in *in vivo* studies [143,144]. mGreenLantern is brighter than either EGFP or EYFP [145]; however, it is less photostable than EGFP and certain other green fluorescent proteins [146,147]. StayGold exhibits high photostability and brightness in both dimeric and monomeric forms [148], while its derivative mBaoJin additionally demonstrates enhanced chemical and pH stability compared with mStayGold [149]. When labeling particular subcellular structures, the pH stability of the fluorophore may be critical; in such cases, the orange-red protein mKO1 is used [150].

It should be noted that when pCAG-IG is used as the vector, fluorescent protein expression is weaker than that achieved with a vector lacking an IRES [151]. pCAG-IG is a bicistronic mammalian expression vector containing a strong, constitutive CAG promoter (a hybrid of the cytomegalovirus early enhancer, chicken β-actin promoter, and rabbit β-globin splice acceptor) that drives the co-expression of the gene of interest and an internal ribosome entry site (IRES)-driven green fluorescent protein (GFP) reporter to track transfected cells [152]. In this case, weak labeling of cellular processes may occur; therefore, it is additionally recommended to introduce mVenus (a YFP derivative), a fast-maturing protein that provides superior labeling of cellular processes owing to its penetration into secretory vesicles [153].

Red fluorescent proteins are also employed in a number of experimental settings. They are particularly convenient in “double” electroporation paradigms, where they allow discrimination between populations of neural progenitor cells electroporated on different embryonic days [154,155], or when experiments require transgenic mouse lines that already express a fluorescent protein, such as Gfap-GFP transgenic mice [106].

Among red fluorescent proteins, the following are commonly used. mCherry is a rapidly maturing, photostable monomer [156] and, by virtue of these properties, frequently features in IUE-based studies. FusionRed was developed subsequently and has proven to be a low-toxicity red protein, outperforming mCherry in the labeling of cellular structures [157]. The more recent mScarlet3, which has not yet achieved comparable adoption, combines the highest quantum yield among RFPs with strict monomeric behaviour, an absence of overt cytotoxicity, and rapid, complete maturation, although this has thus far been confirmed only by *in vitro* experiments [158]. One of the first RFPs was DsRed, a naturally occurring tetramer [143,159]. Subsequently, proteins less prone to oligomerization, such as those listed above, came to be preferred. One of the first such monomers was TagRFP, which matured more rapidly and exhibited high brightness relative to the preceding mRFP variants [157,160].

In addition to those discussed here, many other fluorescent proteins are available, including variants emitting in other regions of the spectrum. Their comparative characteristics are summarized in a dedicated review [138].

### 2.5. Selection of the Embryonic Stage for IUE

The embryonic stage (E) at which IUE is performed is chosen according to the cell population to be targeted and the region of interest (more precise regional targeting is achieved through electrode placement):

1. i-GONAD. In this approach, electroporation is performed at the very onset of embryonic development (E0.7), enabling editing of the entire embryo. This may, however, entail certain difficulties—for example, embryonic lethality following complete knockout of some genes, which limits the applicability of the method to such targets [161,162,163].
2. Early embryonic stages (E9.5-E11.5). IUE at these stages is used to deliver plasmid vectors to the retina, midbrain, and thalamus [164,165,166]. Potential limitations should be borne in mind: IUE at E11.5 is associated with lower embryonic survival than at E12.5 [106], and IUE at even earlier stages (E7.5-E8.5) is likewise accompanied by poor embryonic survival [162].
3. Stages E12.5-E16.5. At these stages, DNA is delivered to neural progenitor cells; in mouse embryos, electroporation at E12.5, E13.5, or E14.5 results in transgene expression in pyramidal neurons of cortical layers V/VI, IV, or II/III, respectively [166,167,168,169]. At later stages, coinciding with the onset of gliogenesis (≥E16.5, including postnatal stages), IUE can be used to deliver DNA into glial progenitors, provided that appropriate cell-type-specific promoters are employed [164,170,171].

These features of neurogenesis allow one to target neuronal populations born at different stages of neurogenesis by performing “double” electroporations, in which DNA is delivered on different days of development. This experimental design permits assessment of independent groups of neurons born at different stages of neocortical development in the same embryo [154,155]. For example, performing electroporations five days apart—at E10.5 and again at E15.5—allows layer II/III neurons to be distinguished from deep layer neurons in the same brain [172].

### 2.6. Anaesthesia Protocols

Two approaches are available for the anaesthesia: inhalational and injectable. The inhalational route offers several advantages, including precise control of anaesthetic depth, less pronounced cardiopulmonary depression, and more rapid postoperative recovery [172]. The most widely used inhalational anaesthetic is isoflurane; its concentration is regulated by a vaporizer system, with 4–5% used for induction and 1.5–2.5% used for maintenance [66,173,174]. The drawbacks of inhalational anaesthesia include the high cost of the requisite equipment, the relative complexity of its installation, and potential occupational hazards to the operator in the event of technical failure [175]. Nonetheless, this is the most widely adopted approach.

Injectable anaesthesia is an alternative. It is simpler to implement, as it requires neither specialized equipment nor additional operator training. However, it also presents a number of disadvantages, such as poor controllability and the possibility of cardiac and respiratory complications developing several hours after administration [175]. Among injectable agents, a ketamine–xylazine combination at doses of 80–100 mg/kg and 8–10 mg/kg, respectively, is frequently employed [74,176]. Alternative injectable regimens include intraperitoneal administration of dexmedetomidine (0.25 mg/kg) combined with midazolam (6 mg/kg) plus subcutaneous buprenorphine (0.1 mg/kg) or extended-release buprenorphine (buprenorphine-ER, 1 mg/kg) [177]; intraperitoneal alfaxalone (40–80 mg/kg) with xylazine (10 mg/kg) [173]; and a mixture of medetomidine hydrochloride (0.3 mg/kg), midazolam (4 mg/kg), and butorphanol tartrate (5 mg/kg) [178].

Analgesics are administered in addition to anaesthesia in order to ensure animal welfare following surgery and minimize the confounding effects of pain and stress on experimental outcomes [179]. This is particularly important when inhalational anaesthetics are used, as these agents lack analgesic properties [180]. Accordingly, buprenorphine (0.05 mg/kg body weight) is administered one hour before surgery; three further doses of buprenorphine (0.05 mg/kg) are given beginning 8 h postoperatively, followed by four additional doses at 12-h intervals [72,181]. Alternatively, dexamethasone (2 mg/kg, s.c.) may be administered at the end of the procedure [182].

### 2.7. Preparation of Plasmid DNA and Injection Parameters

The injection mixture for IUE is prepared in sterile water or, less commonly, in phosphate-buffered saline, supplemented with 0.01–0.5% of a tracer dye to allow visualization of the injectate during the procedure [34,73]. When selecting a dye, its potential cytotoxicity must be taken into account. Fast Green, for example, is regarded as non-toxic and does not interfere with embryonic development, whereas many other compounds—such as Trypan Blue—are teratogenic and may cause embryonic death [183,184]. The total plasmid DNA concentration in the mixture should fall within the range of 1–5 µg/µL [74,185]. A concentration of 1 µg/µL is generally considered optimal; however, depending on the activity of the chosen promoter and on the size and stability of the encoded protein, this value may be adjusted empirically [186]. Crucially, the plasmid DNA used for the injection mixture must be high-quality and strictly endotoxin-free, as the presence of even trace amounts of this substance reduces post-electroporation embryo survival [187].

Approximately 0.5–2 µL of the plasmid DNA mixture [188,189,190,191] is injected into the lateral ventricle using a glass capillary, the tip of which should preferably be bevelled at an angle of 35–45° [155,192]. To ensure uniform filling of the ventricle, a microinjector is recommended to be employed with the following settings: injection pressure (pi) = 250–300 hPa; injection time (ti) = 0.8–1.2 s; and compensation pressure = 10–50 hPa [34,72].

### 2.8. Electrode Placement and Electric Field Parameters

The electric field is generated using tweezer-type electrodes (circular platinum paddles), whose diameter should be selected according to the embryonic stage and the desired size of the targeted area. Electrodes ranging from 0.5 to 5 mm in diameter may be used. Smaller paddles provide greater precision in restricting electroporation to the region of interest. In the early stages (E12.5 and earlier), 0.5–1 mm electrodes are recommended because of the small embryo size [191,193]. Four to five square-wave pulses are delivered through the electrodes at a frequency of one pulse per second (50-ms pulse duration followed by a 950-ms interval). This protocol has been identified as the most effective in terms of both DNA uptake and cell survival [74,185,194,195]. The applied voltage should be adjusted according to embryonic age and strain; for example, the literature reports the following parameter values for the CD1 mouse line: E11.5—22 V; E12.5—30 V; E13.5—40 V; E15.5—45 V [73]. A fixed voltage (35 V) irrespective of developmental stage has nonetheless also been applied successfully [181]. For the C57BL/6 strain, lower voltage settings have been recommended: 33–35 V for E12.5-E13.5, 36–38 V for E14.5, and 38–40 V for E15.5 [191]; effective application of 45 V starting from E13.5 has also been reported [196].

Additionally, the CD1 strain has been reported to produce larger litters and to exhibit superior postnatal maternal care compared with C57BL/6J, which may be a relevant consideration when selecting a mouse strain [33,197].

The positioning of the tweezer-type electrodes depends on the targeted transfection area, as described in detail elsewhere [192]. By varying the angle of electrode placement, cells can be transfected in different regions, including the cortex, hippocampus, pituitary gland, and several other structures. Targeting errors occur in no more than 30% of transfections. For instance, to target the hippocampus, the cathode was placed laterally and the anode medially relative to the developing hippocampus [106]. *In utero* electroporation of the ganglionic eminences is also employed. Since dysregulation of inhibitory neurons or their fate can underlie intellectual disability or neuropsychiatric disorders, IUE is used to study the role of genes involved in the development of these neurons [198,199,200] as well as in the regulation of their migration [201]. Furthermore, electroporation of the ganglionic eminences is applied to elucidate the origin of oligodendrocyte progenitor cells [202].

A modification of the tweezer-type electrode incorporating a third additional electrode enables more precise targeting of the region of interest, allows simultaneous electroporation of both brain hemispheres, and permits a change in the targeted region not by repositioning the electrodes but by reversing their polarity [203,204]. Modifications integrating both needle and paddle electrodes into a triple- or quadruple-electrode probe have also been developed. The combined use of needle electrodes is reported to increase electroporation accuracy, and, with sufficient operator experience, embryo survival remains high—86.5% for the tripolar probe and 78.1% for the tetrapolar probe. The trident probe showed an 87.5% fluorescence-positive rate, and the fork probe an 81.3% fluorescence-positive rate, both considerably higher than the rates for tweezer-type bipolar probes and comparable to the previously reported paddle-type tripolar probe [33].

### 2.9. Principal Surgical Risks and Strategies for Their Mitigation

Several complications may arise during surgical procedures in animals that can compromise the validity of experimental results.

There is a risk of intraoperative death of the dam, most commonly associated with severe intra-abdominal haemorrhage and/or prolonged anaesthesia. Excessive pressure on major blood vessels should therefore be avoided [205], and the total duration of surgery should not exceed 30 min [72]. Hypothermia may also develop in the dam during anaesthesia; to maintain body temperature, the animal is placed on a heating pad, and the uterus is regularly moistened with warm saline, which additionally prevents tissue desiccation. Furthermore, to avoid stress caused by excessive corneal drying, ophthalmic gel or artificial tears are applied to the eyes [72,182].

Even when these recommendations are followed and the procedure is technically successful, embryonic survival may be reduced owing to a combination of factors. Chief among these are technical aspects of the IUE procedure itself, most notably the duration of anaesthesia [34] and excessive stretching, twisting, or deformation of the uterine horns, which compromises embryonic blood supply [172]. Appropriate electroporation parameters and correct orientation of the tweezer-electrodes relative to the embryos are likewise of considerable importance. Contact with the placentas and with the dorsal, highly vascularized surface of the uterus should be avoided, and electrodes should not be positioned over these structures so as to prevent cauterization of the vessels supplying them [172,206].

Rupture or partial damage of the amnion constitutes a further risk factor. This may result either from excessive compression of the embryo by the tweezer-electrodes during IUE or from the use of inappropriate capillaries. For example, the capillary tip may have insufficient sharpness or an excessively large tip diameter [155]. Before injection, it is essential to verify that the microcapillary tip is open and that a moderate outflow of the dye-labeled plasmid DNA solution occurs [155]. Improper microinjection technique leads to the formation of pseudo-heterotopias and pseudo-ectopias at the injection site [196]. To prevent such artifacts, high-quality capillaries bevelled at 35–45° are recommended [155,192]; the puncture depth should be approximately 1 mm, and the injected volume should not exceed 2 µL (exceeding this volume at E14.5 may result in hydrocephalus [155]). It is essential to maintain a consistent volume of injected plasmid mixture across all embryos, since even minor variations in injection volume can affect the degree of transfection, the size and localization of the electroporated area, and consequently the reproducibility of the resulting phenotypes [72]. In addition, disruption of brain integrity caused by IUE can lead to abnormal microglial migration, local inflammation, and degeneration of radial glia. The long-term consequences of these effects remain unclear [207,208].

Such artifacts can be identified using specific markers: DAPI (visualization of tissue “bulges”), Tuj1 (premature differentiation), Ctip2/TBR1 (disruption of laminar organization), laminin (basement membrane breaches), nestin (radial glia disruption), and reelin (disruption of the marginal zone) [196,205].

When processing the biological samples, one may encounter the problem of low electroporation efficiency—a small number of fluorescent cells or their complete absence. In such cases, the cause may lie either with the electroporator (insufficient output voltage) or with faulty electrodes. To diagnose this, it is recommended to additionally monitor the output current parameters displayed on the device’s control panel during pulse delivery. To verify electrode functionality, a test pulse should be delivered in PBS: bubble formation should be observed in the solution with each pulse. Cleaning the electrodes with ethanol should be avoided, as this may damage the platinum surface and reduce its conductivity, thereby further compromising the efficiency of the method [172]. A further limitation of IUE concerns the reproducibility of experimental data. In particular, Russell J. et al. emphasize that many studies lack an explicit description of section-matching procedures across independent electroporations as well as the use of littermate controls [115]. Nevertheless, statistical analyses in such studies are still commonly based on a limited number of sections obtained from a small number of embryos (3–4) across a few litters (2–3) [209,210].

In addition, Russell J. et al. emphasize that the interpretation of experimental results strongly depends on the quality of controls, including transfection efficiency, targeting accuracy, and the degree of mosaicism. To address this issue, the authors developed a novel plasmid vector, “Double UP,” previously described by us, which incorporates two fluorescent proteins to provide a built-in internal control, as well as an automated software tool, “TRON,” for the analysis of neuronal migration, enabling a more objective assessment [115].

Another significant factor determining the downstream interpretation of results is the efficacy of the constructs themselves, as the visualization of reporter proteins (GFP, RFP, etc.) merely reflects vector delivery. Several approaches can be employed to address this issue. In particular, CRISPR/Cas9-mediated gene inactivation can be confirmed by DNA sequencing and/or Western blot analysis [211]. The functionality of shRNA can likewise be verified by Western blot or RT-PCR/qPCR [212]. An additional confirmation may be provided by immunofluorescence analysis using antibodies against the protein targeted by the DNA/RNA-based inactivation system [213].

Thus, the IUE method offers a number of advantages over other approaches to plasmid vector delivery, and its “flexibility”, which allows the specified parameters to be modified, makes it a promising tool for studying the developmental genetics of the cerebral cortex. With sufficient operator experience and adherence to all recommendations, post-operative embryo survival is reported to range from >80% to 95%, while electroporation efficiency (the proportion of embryos expressing fluorescent protein from the electroporated construct) exceeds 90–98% [73,196,197]. However, it also has a number of features that must be taken into account when planning an experiment.

## 3. Application of Thymidine Analogues in Studies of Early Neurogenesis

The development of the mammalian brain requires precise spatiotemporal coordination of such processes as the proliferation and differentiation of neural progenitor cells. Each dividing cell progresses through a defined sequence of cell cycle phases, among which two are active: the synthesis phase (S phase), during which DNA replication takes place, and mitosis (M phase), i.e., division of the cell itself. In addition, three further phases are distinguished: the quiescent phase (G0), in which cells exit the cell cycle, and two preparatory phases—the presynthetic phase (G1), during which cell volume increases in preparation for DNA replication, and the postsynthetic phase (G2), during which replication is completed and preparation for division begins [214]. Changes in the duration of the cell cycle or of its individual phases represent one of the key mechanisms regulating corticogenesis. Thus, in the early stages of embryonic development, a short cell cycle promotes expansion of the neural progenitor pool, whereas the gradual lengthening of G1 leads to cell cycle exit and the onset of differentiation. Over this period, the cell cycle increases from 8 to 18–20 h [74,215]. By the end of embryonic development, most progenitors have exited the cell cycle, and neurogenesis in the VZ is essentially complete. For this reason, determination of the duration of the total cell cycle (Tc) and of its individual phases is used to assess the balance between proliferation and differentiation in the developing cerebral cortex [216,217,218].

To investigate cell proliferation *in vivo,* synthetic thymidine analogues are frequently employed; these are incorporated into the DNA molecule during its replication in the S phase of the cell cycle. This results in irreversible labeling of cells that were undergoing DNA synthesis at the moment the label was administered. In combination with immunohistochemical methods and specific cell cycle markers, these analogues are widely used to study cell cycle kinetics, to determine the duration of individual phases, to analyse cell cycle exit and to assess neurogenesis both under normal conditions and in various pathologies [219].

### 3.1. Thymidine Analogues

The most commonly used synthetic thymidine analogues are 5-bromo-2′-deoxyuridine (BrdU), 5-ethynyl-2′-deoxyuridine (EdU), 5-chloro-2′-deoxyuridine (CldU) and 5-iodo-2′-deoxyuridine (IdU). All of them share the same principle of action but differ in the mode of detection and in the scope of their application [220].

BrdU has long remained the most widely used analogue owing to the high reproducibility of the results obtained, the large number of established experimental protocols and the possibility of combining it with various methods of immunohistochemical analysis. Its principal drawback is the need for prior DNA denaturation to allow antibody binding. Most commonly, BrdU epitope unmasking is achieved by treating the tissue with hydrochloric acid [221] or formamide [222]. Heat-induced epitope retrieval in citrate or Tris-EDTA buffer is also used [223]. However, inappropriate application of these approaches may compromise tissue preservation and reduce the efficiency of staining for certain protein markers [220].

In contrast to BrdU, EdU is detected by means of a “click chemistry” reaction, which does not require DNA denaturation. This substantially shortens the analysis time, ensures better preservation of tissue morphology and facilitates multiplex immunofluorescence staining [224]. The analogues CldU and IdU are used predominantly for sequential or dual labeling of cells. Their use makes it possible to distinguish several successive “waves” of DNA synthesis, to determine neuronal birthdates, to detect cell cycle re-entry and to characterize the features of S-phase progression. However, the simultaneous use of several thymidine analogues requires careful selection of antibodies, since cross-reactivity between halogenated thymidine derivatives is possible [224].

The concentrations of thymidine analogues administered to pregnant mice vary considerably depending on the aims of the study, the administration schedule and the experimental model. In studies of mouse corticogenesis, BrdU is most frequently used in the range of 50–200 mg/kg and EdU at approximately 20 mg/kg, whereas for dual labeling, CldU and IdU are usually administered at 50 mg/kg each. It should be noted that exceeding the recommended doses or performing repeated administrations, particularly of EdU, may be accompanied by cytostatic and cytotoxic effects, which must be taken into account when designing experiments [220].

### 3.2. Cell Cycle Analysis Using Thymidine Analogues

BrdU injections are widely applied both on their own—to characterize the phenotypes of animal models and to assess the birthdates of individual cell populations and their subsequent fate—and in combination with IUE for a detailed analysis of cell cycle dynamics and phases.

Quantitative assessment of the results obtained after immunohistochemical staining of sections for BrdU is best performed using ImageJ (Fiji) 2.18.0/1.54p (Fiji project, MPI-CBG, Dresden, Germany) software together with the Cell Counter plugin, which ensures reproducible manual counting of cell populations on confocal images. To obtain reliable data, confocal imaging should be carried out in Z-stack mode with a step of 1–1.5 µm [75,225] covering the entire thickness of the cortical region of interest, and the acquisition parameters must be identical for all samples. The chosen Z step is optimal and prevents the loss or double-counting of individual cells when switching from one optical plane to another.

**Figure 4:**
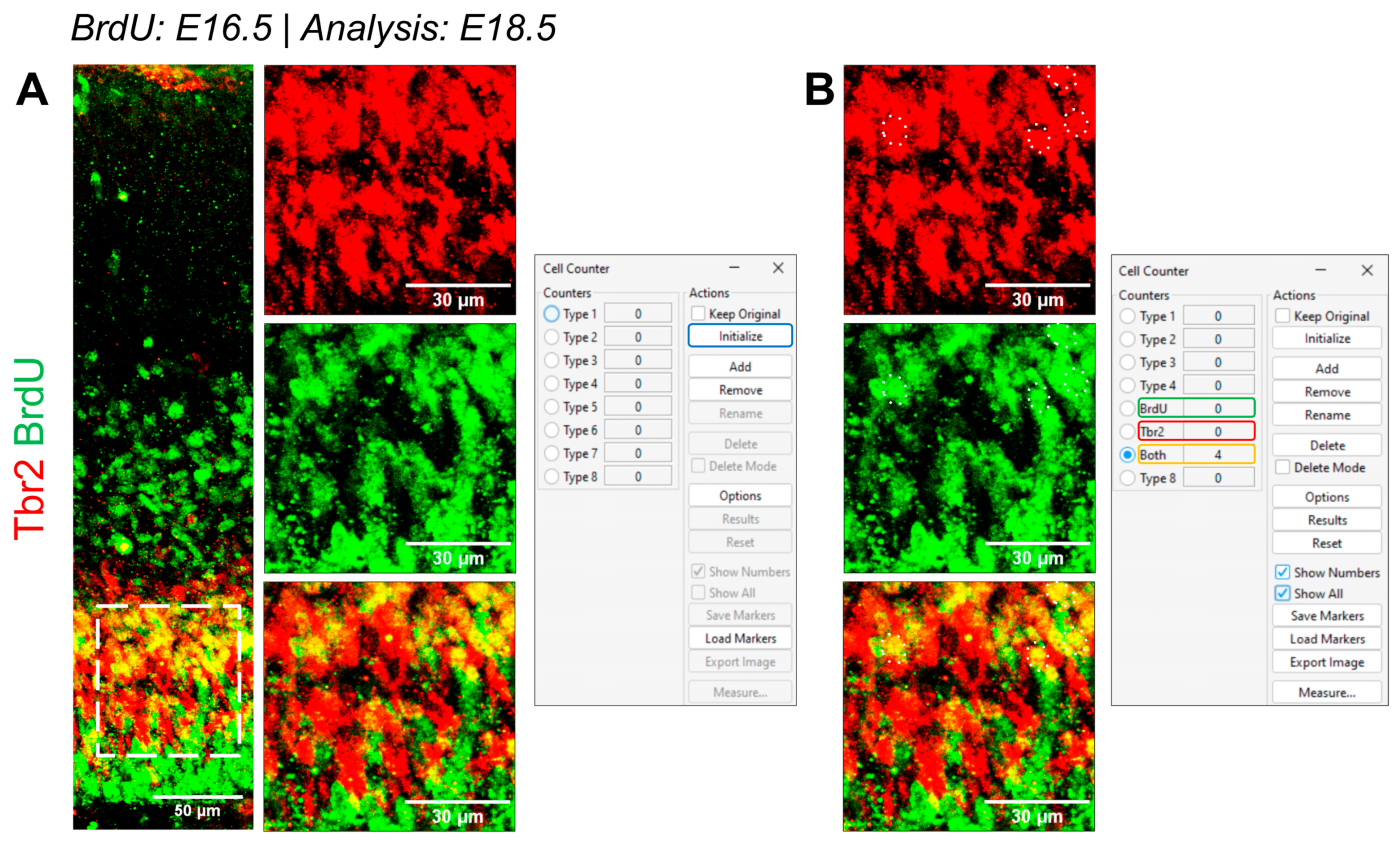
Labeling and counting of neural progenitor cells and their subpopulations in ImageJ (Cell Counter). (**A**)—Selection of region of interest and initialization of Cell Counter; (**B**)—manual annotation of BrdU^+^ and Tbr2^+^ electroporated cells and their subpopulation. Green channel: detection of BrdU using Alexa Fluor 488 secondary antibodies (Ex 488 nm, Em 510–530 nm). Red channel: detection of Tbr2 using Alexa Fluor 647 secondary antibodies (Ex 647 nm, Em 665–685 nm). Created in Microsoft PowerPoint 2024 (Microsoft Corporation, Redmond, WA, USA). Adapted from previous work [226].

As an example, we may consider the analysis of coronal mouse brain sections after BrdU administration at E16.5 and subsequent immunohistochemical analysis at E18.5. Confocal Z-stacks (step size, 1–1.5 µm) are opened in ImageJ, an ROI is delineated, and the Cell Counter plugin is used (Figure 4A) to count BrdU^+^, Tbr2^+^, and double-positive cells (Figure 4B). The plugin allows cells to be marked in different channels and the output file to be saved for later re-overlay without recounting.

This approach was successfully used by us to assess the birth time of Tbr2^+^ cells upon inactivation of the NeuroD2/6 transcription factors. Using BrdU pulse labeling, it was shown that the majority of ectopically localized Tbr2^+^ cells in the brains of double knockout mice are generated after the E14.5 stage [226].

**Figure 5:**
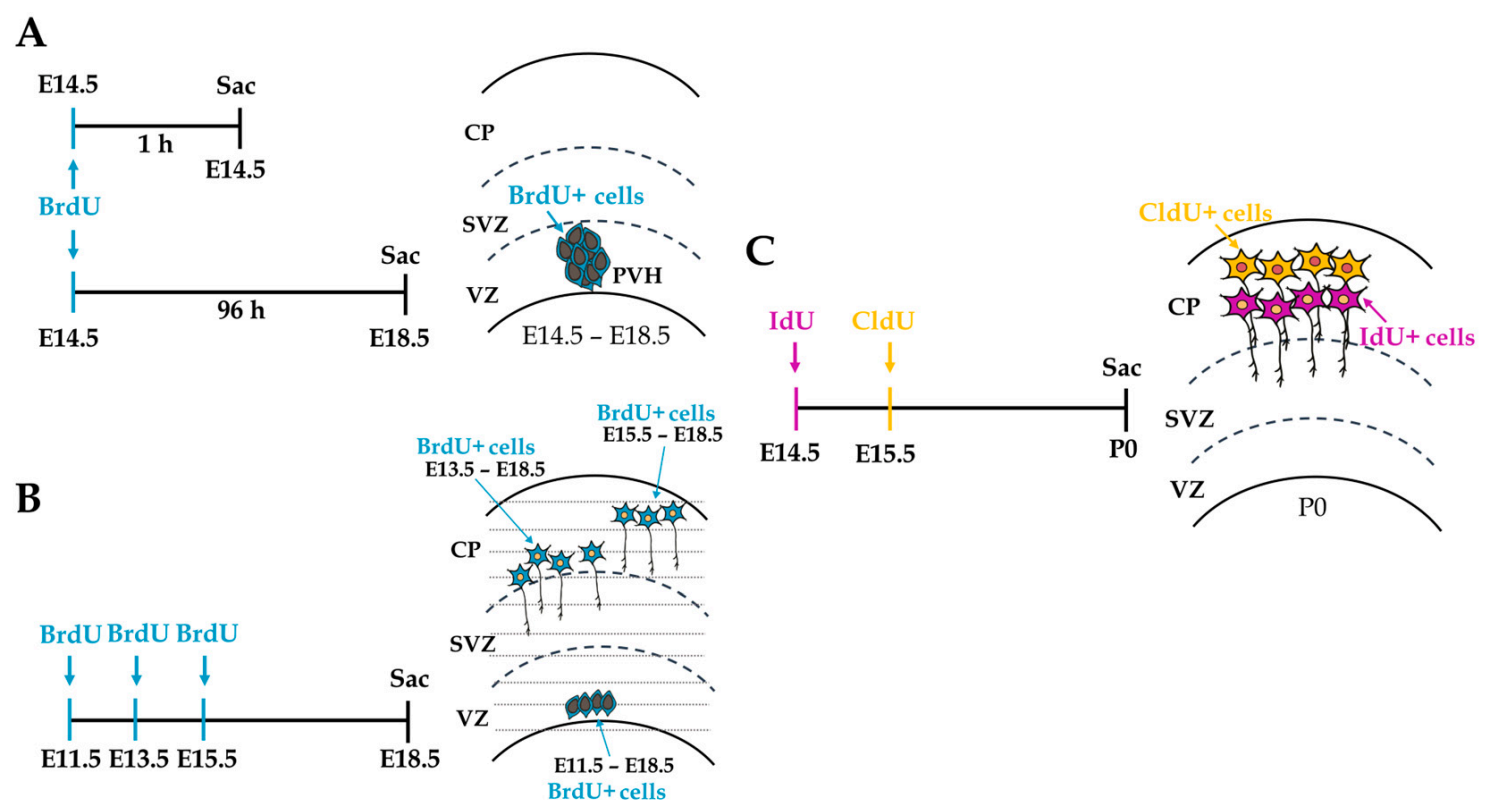
Schematic representation of the experimental application of thymidine analogues. (**A**)—analysis of the timing of PVH formation by varying the duration of BrdU exposure; (**B**)—analysis of the migration of cell populations born at different embryonic stages; (**C**)—analysis of the migration of progenitor cell populations by means of sequential labeling with different thymidine analogues. E—embryonic day, P—postnatal day, sac—sacrifice, h—hour, VZ—ventricular zone, SVZ—subventricular zone, and CP—cortical plate. Created in Inkscape 1.4.2 (The Inkscape Team, Boston, MA, USA).

In the work Sarkisian et al., a short BrdU exposure (1 h) at stage E14.5 was used to determine the number of neural progenitor cells located in the VZ and residing in S phase during the formation of periventricular heterotopia (PVH) [227]. A longer exposure (96 h) with analysis at E18.5 showed that BrdU^+^ cells that had exited the mitotic cycle were still located within the PVH and had not migrated into the CP. A similar series of experiments was performed at stage E18.5, which made it possible to establish the approximate timing of PVH formation during embryonic development—at E14.5 or earlier (Figure 5A). It is important to keep in mind that BrdU content in a nucleus will be diluted with every cell cycle until it becomes undetectable. This means that cells with detectable BrdU levels either left mitotic cycle or underwent very few cell divisions after they have incorporated BrdU.

Labeling with thymidine analogues also makes it possible to assess the distribution of neurons born at different stages of corticogenesis. Thus, in the study by Hui Wang et al., BrdU was injected at different stages (E11.5, E13.5 and E15.5) with subsequent analysis at E18.5 [228]. The confocal images obtained were divided into 10 equal bins spanning from the VZ to the CP, and the number of BrdU^+^ cells was counted in each bin. Using this approach, it was demonstrated that inactivation of *Gli3* disrupts laminar distribution (Figure 5B).

In the study by Nakanishi et al., BrdU was injected at stages E14.5 and E15.5 with analysis at P0, and in the confocal images, the cerebral cortex was divided into 8 equal bins from the VZ to the MZ [178]. There are also studies in which images were divided into 5 equal bins, nominally corresponding to the ventricular zone (VZ), subventricular zone (SVZ), intermediate zone (IZ), and the lower and upper portions of the cortical plate (CP) [74,169].

In the work of F. Pérez-Martínez et al., transgenic nestin-reelin mice, in which reelin is expressed under the control of the nestin promoter and thus ectopically in neural progenitor cells, were administered CldU and IdU at stages E13.5 and E14.5, respectively, with analysis at P0 (Figure 5C) [229]. Dual labeling demonstrated that later-born progenitors (IdU^+^) migrate in an “inside-out” manner past the earlier-born population (CldU^+^), thereby partially recapitulating normal neurogenesis upon restoration of reelin expression levels in this transgenic mouse line. An analogous approach combining thymidine analogues (BrdU/EdU) to label neural progenitor cells at different stages of embryonic development has been used in a number of studies [178,230].

A further extension of this strategy is its combination with IUE, which makes it possible to analyse cell cycle parameters, migration and subsequent fate of the transfected population of neural progenitor cells, which is particularly important for the functional study of gene functions *in vivo.*

This approach yields the total number of electroporated cells and allows them to be assigned to subgroups according to marker expression, after which the proportions of the corresponding populations (as percentages) relative to the total number of GFP^+^ cells in the analyzed region can be calculated. In particular, it is possible to quantify the proportion of GFP^+^ cells that have passed through S phase (GFP^+^/BrdU^+^), that retain proliferative activity (GFP^+^/BrdU^+^/Ki67^+^), that have exited the cell cycle (GFP^+^/BrdU^+^/Ki67^−^), and so forth. Examples of studies in which the combination of IUE and thymidine analogue labeling has been used to analyse cell cycle phases, the birthdates of neural progenitor cells, and the features of their migration are given below.

One of the studies that demonstrated in detail the potential of BrdU labeling in combination with IUE was that of Stancik et al. [74]. The authors showed that the transfection of neural progenitor cells in the VZ is neither uniform nor random: most of them were in M phase at the time IUE was performed, and the further a cell was from the S–M phases, the lower the probability of its transfection.

**Figure 6:**
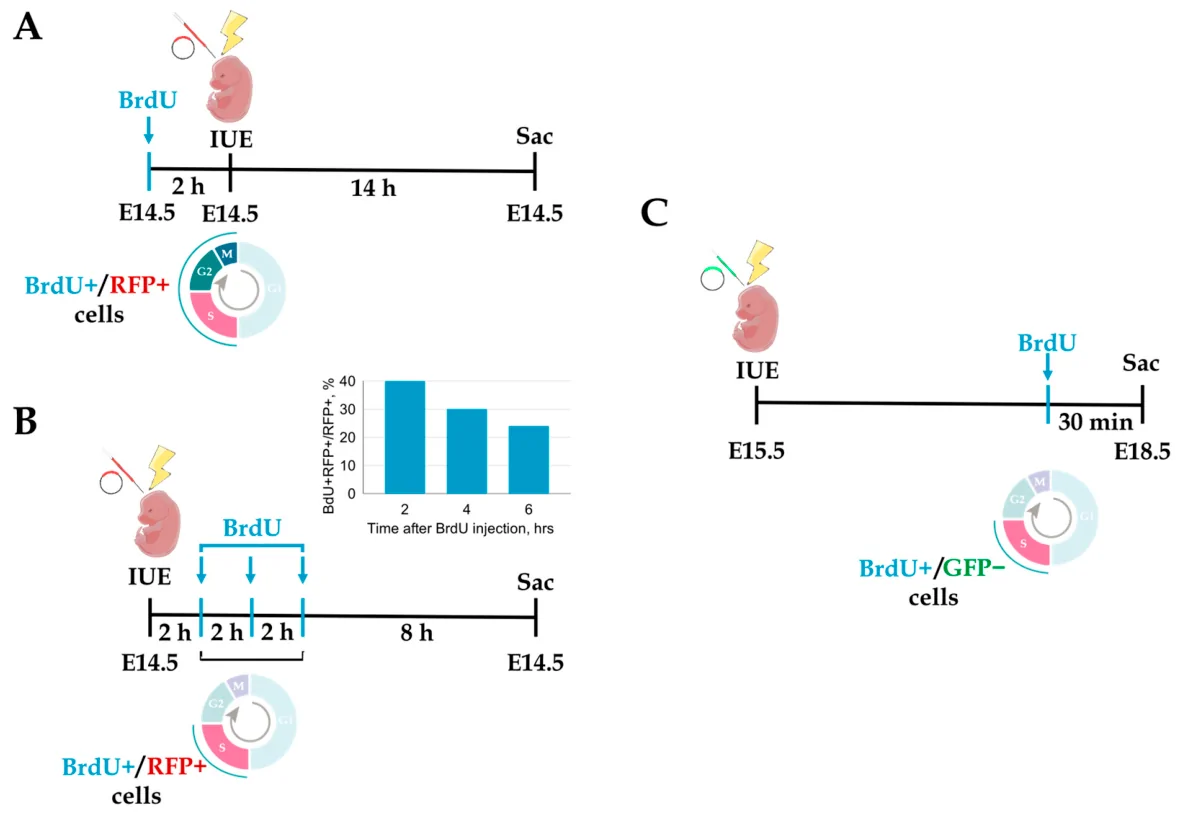
Schematic representation of the experimental application of thymidine analogues in combination with IUE. (**A**)—labeling of progenitor cells in S phase 2 h before IUE; (**B**)—labeling of cells with a thymidine analogue 2, 4 and 6 h after IUE; (**C**)—labeling of cells in S phase 30 min before brain collection. E—embryonic day, P—postnatal day, sac—sacrifice, and h—hour. Created in Inkscape 1.4.2 (The Inkscape Team, Boston, MA, USA).

To confirm this hypothesis, the authors combined IUE of the plasmid vector pCAG-RFP with BrdU injections. Since BrdU is incorporated into cells in S phase, varying the timing of its administration relative to the moment of electroporation made it possible to determine the cell cycle phase in which transfected cells had been at the time of electroporation. For instance, a single BrdU administration 2 h before IUE labeled neural progenitor cells (BrdU^+^) that were in the S–M phases at the time of IUE (Figure 6A), whereas administration of BrdU immediately after IUE labeled only cells in S phase. With subsequent interval injections (every 2 h), the labeled cells were those in S phase at the given interval after IUE. Fourteen hours after IUE, double-positive RFP^+^/BrdU^+^ cells were quantified within the VZ (100 × 100 µm), and the proportion of double-positive RFP^+^/BrdU^+^ cells relative to the total number of RFP^+^ cells in the same region was calculated. The following relationship was demonstrated: as the interval between electroporation and subsequent BrdU administration increased, the proportion of double-labeled cells progressively decreased (Figure 6B). This reflected the limited temporal window of effective transfection, restricted to the S–M phases of the cell cycle.

In the work of Langevin et al., the tendency of early neural progenitor cells to populate the deep cortical layers was confirmed [169]. To this end, on the day of IUE with the plasmid vector pCIG2-GFP, BrdU was also injected at different stages (E12.5, E13.5, E14.5 and E15.5), while the stage of analysis remained constant, at E18.5. Comparative analysis was based on the position of GFP^+^ cells relative to BrdU^+^ cells. In addition, BrdU injection was used to confirm that GFP-positive cells were postmitotic by stage E18.5. For this purpose, BrdU was administered 30 min before tissue collection at E18.5 following electroporation at E15.5. The absence of BrdU^+^/GFP^+^ cells indicated that the transfected cells were not proliferating at the time of BrdU administration (Figure 6C).

### 3.3. The Use of “Markers” to Determine Cell Fate After Thymidine Analogue Injection

The principal drawback of labeling with BrdU and other thymidine analogues remains the non-selective nature of the labeling, which precludes discrimination between individual populations and determination of the subsequent fate of transfected neural progenitor cells. For selective analysis in such cases, markers specific to particular processes and/or cell populations are used [28,231].

To assess the proliferation of neural progenitor cells, immunohistochemical analysis of BrdU/EdU is combined with the proliferation marker Ki67. Whereas thymidine analogues are incorporated into the DNA of cells that are in S phase at the moment of label administration, Ki67 is expressed in all active phases of the cell cycle (G1, S, G2 and M) and is absent from cells that have entered quiescence (G0). The combination of these markers makes it possible to determine whether a cell is still proliferating or has already left the cell cycle at the time of tissue collection for subsequent analysis [196,232,233].

**Figure 7:**
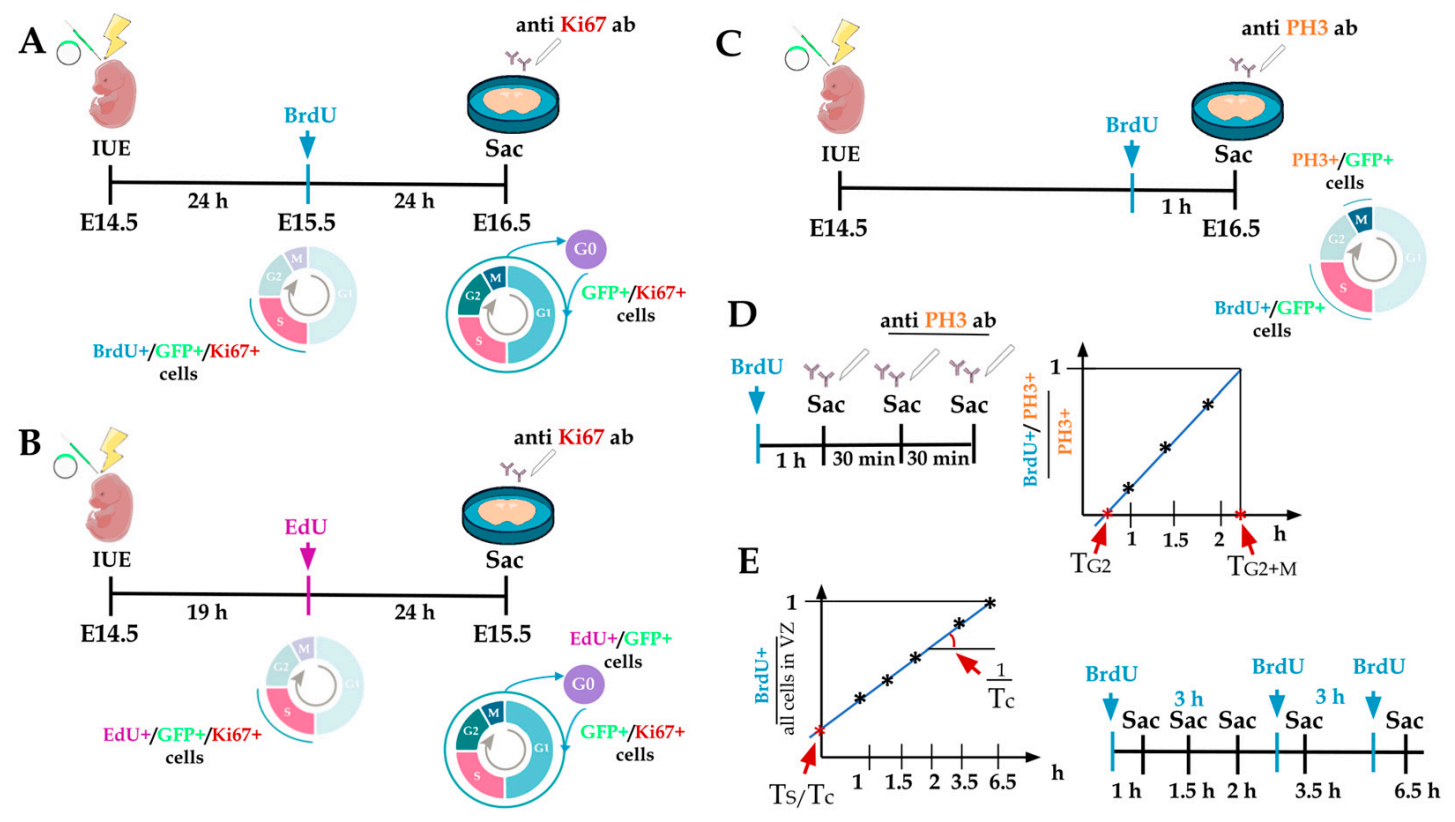
Schematic representation of the experimental application of thymidine analogues in combination with IUE and cell cycle markers. (**A**)—interpretation of cell fate after BrdU injection; (**B**)—assessment of progenitor cell cycle re-entry after EdU injection; (**C**)—labeling of cells with BrdU or PH3 for the analysis of division rate and nuclear position, respectively; (**D**)—calculating G2/M phase durations using BrdU injections; and (**E**)—calculating the duration of the cell cycle by cumulative labeling with BrdU. E—embryonic day, P—postnatal day, sac—sacrifice, h—hour, and ab—antibodies. Created in Inkscape 1.4.2 (The Inkscape Team, Boston, MA, USA).

The BrdU^+^/Ki67^+^ population is defined as cells continuing to proliferate after incorporation of the thymidine analogue, whereas BrdU^+^/Ki67^−^ cells are considered to have entered differentiation [234]. However, it should be noted that confirming the transition of neural progenitor cells to the differentiation stage requires the use of specific markers for neuronal populations at subsequent stages of developing cortical zone formation, such as Tbr2, Tbr1, and others. An analogous approach to the interpretation of cell fate is used in conjunction with IUE. Thus, inactivation of *Spout1* leads to retention of proliferating progenitors within the cell cycle. BrdU was injected 24 h after IUE performed at stage E13.5, and tissue was collected at E15.5. The presence of BrdU^+^/Ki67^+^/GFP^+^ and Ki67^+^/GFP^+^ populations indicates that the transfected cells were in S phase of the cell cycle 24 h after IUE, at the time of BrdU injection, and that a further 24 h later, they were still in a proliferative state (Figure 7A) [235].

In another study, to assess cell cycle re-entry, EdU was administered 19 h after IUE performed at stage E14.5, with subsequent analysis 24 h after injection. The EdU^+^/Ki67^+^/GFP^+^ cell population remains in a proliferative state after EdU incorporation, whereas EdU^+^/GFP^+^ cells represent differentiated neural progenitor cells (Figure 7B) [236].

Furthermore, the combination of IUE, BrdU and Ki67 can be used to investigate the cell cycle exit index. This was demonstrated in the work of Takahito Yumoto et al. [237]. Following IUE at stage E14.5, pregnant females were administered BrdU daily from E15.5 to E17.5, and analysis was performed at E17.5. The cell cycle exit index was calculated as Equation (1):

cell cycle exit index = [(number of GFP^+^/BrdU^+^ cells) − (number of GFP^+^/BrdU^+^/Ki67^+^ cells)]/(total number of GFP^+^/BrdU^+^ cells) (1)

The higher its value, the greater the number of proliferating neural progenitor cells that have progressed to further differentiation.

The combination of BrdU with subsequent immunohistochemical staining for phosphorylated histone H3 (PH3) is used to identify neural progenitor cells in M phase. A proportion of the cells labeled with BrdU in S phase progresses to G2 phase and mitosis and will consequently be labeled as PH3^+^ [238].

Using PH3, the duration of the G2/M phases can be determined as follows: brains are collected 1, 1.5 and 2 h after a single BrdU injection; the ratio of BrdU^+^/pH3^+^ cells to the total number of pH3^+^ cells in the VZ is plotted; and the point at which the resulting line intersects the x axis indicates the duration of G2 [234]. The duration of G2+M is measured as the minimum time required for all mitotic cells (PH3^+^) along the ventricular surface to become BrdU-labeled (BrdU^+^) (Figure 7D) [239].

In addition, the duration of the cell cycle and of its individual phases can be calculated by means of cumulative labeling with BrdU or other thymidine analogues. The principle of the method is based on repeated BrdU administration at defined time intervals—for example, 3 h between injections—with embryonic brains collected 1, 1.5, 2, 3.5, 6.5, 15.5 and 24.5 h after the first administration. In the resulting samples, the labeling index (the ratio of BrdU^+^ cells to the total number of cells) is determined. The data obtained are extrapolated to the point of intersection with the Y axis, the value of which represents the ratio of S-phase length to total cell cycle duration (Ts/Tc), while the reciprocal of the total cell cycle length (1/Tc) is calculated from the slope of the cumulative curve (Figure 7E) [215,234].

By varying the BrdU administration schedule and the time of biological material collection, it is possible to determine the rate of transition between individual cell cycle phases and to assess the impact of genetic manipulations on the proliferative activity of cells and on phase durations. Thus, the position of the nuclei of GFP^+^/BrdU^+^ or PH3^+^ cells after IUE at stage E14.5 was used to study changes in the rate of cell division and in the position of neural progenitor cell nuclei over the course of the cell cycle. BrdU was injected 1 h before tissue collection at E16.5 (Figure 7C) [240]. PH3^+^ staining is also used to delineate the VZ and SVZ. In one series of experiments, the stage of electroporation remained constant (E12.5), while the time of analysis was varied, amounting to 6, 9, 12, 24, 48 and 72 h and 6 days after IUE. The position of PH3^+^ cells was assessed in the analyses performed at 6 and 9 h: apical mitoses delineated the ventricular zone, and basal mitoses delineated the subventricular zone. On the basis of the boundaries thus defined, it was shown that GFP^+^ cells were predominantly located in the VZ at 6 h, whereas at 9 h and later an increasing number of cells had migrated towards the SVZ and subsequently into the CP [169].

As a complementary approach, TUNEL analysis (terminal deoxynucleotidyl transferase dUTP nick end labeling) may be applied to assess cell death at different stages of corticogenesis [234,239,241].

Thus, the combination of IUE with intraperitoneal injection of thymidine analogues and subsequent immunocytochemical analysis of certain markers makes it possible both to calculate the length of the cell cycle, including that of each of its individual phases, and to determine the fate of the neural progenitor cell—that is, the cell cycle phase in which the transfected cell was at the moment of thymidine analogue injection and/or IUE, and whether it had progressed to the next stage of differentiation by the time of analysis.

## 4. Methods for the Analysis of Axonal and Dendritic Processes

In this chapter we will focus on the morphological analysis of neurons, and in particular on the assessment of axonal and dendritic development, since the development of these structures is one of the fundamental aspects of neural network formation [242].

### 4.1. Neuronal Polarisation

One of the important stages of neuronal development is neuronal polarisation, which is an intermediate step between early differentiation and subsequent cell migration. It begins with formation of two processes on the opposite poles of a cell: an axon and a leading process. The leading process moves ahead of the cell body during migration. After migration is completed, it will transform into an apical dendrite [243].

IUE allows both nascent axon and leading process to be visualized in the developing cortex [244]. One approach to assessing polarisation is based on the analysis of the subcellular localisation of organelles, in particular the Golgi complex.

Thus, to identify the role of the IGF-1R (shIGF-1R-DsRed) in neuronal polarisation, IUE was performed at stage E15.5 with the addition of a construct containing the Golgi enzyme Gal-T2 together with YFP. In the analysis at stage E17.5, the Golgi complex was considered not to be oriented towards the cortical plate if the majority of the Gal-T2-YFP fluorescent signal was concentrated towards the ventricular surface (in the lower part of the horizontal cell axis) rather than in the apico-lateral direction [245]. According to the results, 60% of electroporated cells with *IGF-1R* knockdown displayed random orientation of the Golgi complex, which was accompanied by disrupted polarity and an inability to form an axon [246].

A similar experimental design employing fluorescent labeling of organelles in neuronal cells was applied in the work of Keiko Nakanishi et al. in a study of SAD-A kinase knockout mice. This kinase is a member of the AMP-activated protein kinase (AMPK) family, which promotes the specification of axon–dendrite polarity through phosphorylation of tau protein in cortical neurons [247]. In this study, IUE was performed in mouse embryos at E14.5 with the plasmid vector pCAG-PACT-mKO1, which encodes the PACT protein that labels centrioles, while the fluorescent protein mKO1 provides their visualisation as an orange signal. Cells migrating in the IZ were analyzed; the position of the centrosome relative to the nucleus of each migrating neuron was determined. Neurons were considered correctly oriented if their centrosomes were located in the upper quadrant of the cell, facing the pial surface [178].

In an auxiliary method for assessing impaired polarisation of migrating neurons, researchers proposed counting the number of leading processes per neuron migrating within the IZ. In addition, the angle of deviation of the leading process relative to the pial surface is measured: cells with a deviation angle of less than 45° [185] or 75° [240] are classified as radially oriented, whereas the remainder are assigned to the non-radial category.

### 4.2. Axon Navigation

Once polarisation is complete, mature neurons form axonal projections to their respective targets, thereby establishing specific connections between different brain regions [248]. To analyse the growth of cortical axons, IUE is applied at early stages of embryonic development, E13.5-E15.5 [213,249]. The orientation of the electrode depends on the target cortical area: when targeting the primary somatosensory cortex (S1) it is applied at an angle of approximately 30–45°, whereas for the primary motor cortex (M1) it is applied at an angle of 0° [250]. Subsequent analysis of axonal projections may be carried out both at later embryonic stages and in the postnatal period.

**Figure 8:**
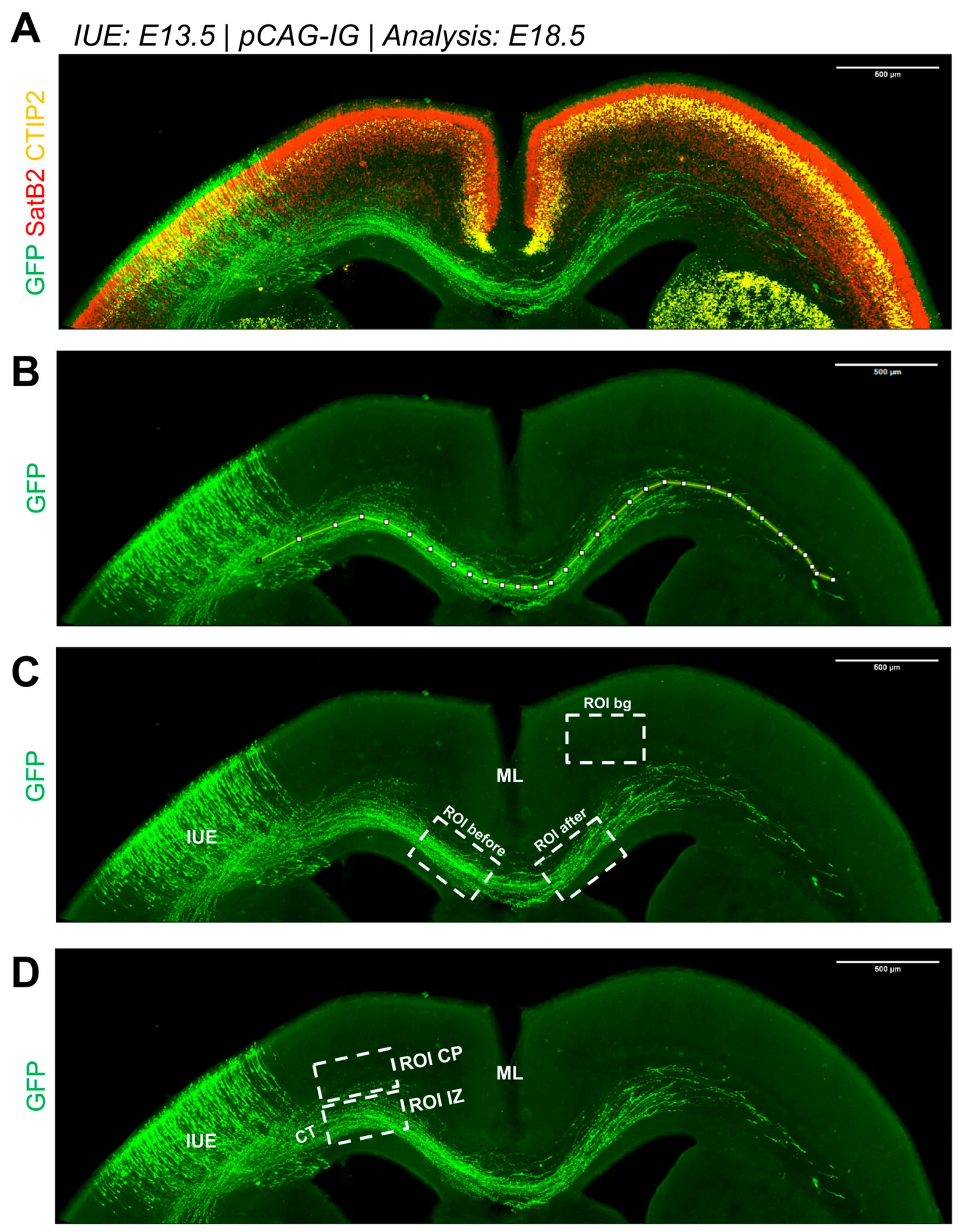
Quantitative analysis of fasciculation and outgrowth of callosal axons after IUE. (**A**)—Representative coronal section at E18.5 showing GFP-positive neurons and laminar markers Satb2/Ctip2. (**B**)—Quantification of cumulative axonal outgrowth. (**C**)—Assessment of interhemispheric navigation efficiency. (**D**)—Morphometric evaluation of axonal fasciculation. Green channel: detection of EGFP (Ex 488 nm, Em 510–530 nm). Red channel: detection of Satb2 using Alexa Fluor 555 secondary antibodies (Ex 555 nm, Em 565–585 nm). Yellow channel: detection of Ctip2 using Alexa Fluor 647 secondary antibodies (Ex 647 nm, Em 665–685 nm). IUE—*in utero* electroporation. CP—cortical plate; CT—callosal tract; IZ—intermediate zone; ML—midline; ROI—region of interest. Created in Microsoft PowerPoint *2024* (Microsoft Corporation, Redmond, WA, USA). Adapted from previous work [213].

Standardized approaches to image processing are required for the quantitative description of these projections and for comparing phenotypes between experimental groups. The analysis of such characteristics of axonal projections as axon length, midline (ML) crossing and the degree of fasciculation can be performed in the Fiji (ImageJ) application, which has become widely used for the analysis of biological data [251]. As an example, let us consider the assessment of these parameters on confocal images (Z-stack with a step of 0.8–1.0 µm [250,252] of coronal brain sections of C57BL/6 mice at stage E18.5 following IUE with the constructs pCAG-IG and pCAG-Venus performed at stage E13.5. To determine the boundaries of the cortical plate and to detect the electroporated neuronal population, the sections were additionally stained against Satb2/CTIP2/GFP (Figure 8A). For quantitative analysis, Z-stacks were first merged into a single image, and subsequent measurements were performed in the electroporation channel.

To assess axon length, axons are traced manually using the Segmented Line tool, starting from the center of electroporation in the IZ and following the commissural tract to the last visible projection. Length is measured with Analyze → Measure (Figure 8B) after setting the scale. To avoid false positives, sections should be anatomically matched, and the density and region of electroporation should be comparable; somatic GFP signals or ambiguous axonal areas should be excluded.

The midline crossing of callosal axons was assessed by measuring the GFP signal in two symmetrical ROIs of equal size along the axonal tract in the IZ: ROI before (ipsilateral to the ML) and ROI after (contralateral to the ML), together with a background ROI (Figure 8C). Mean intensity was background-corrected as shown in Equation (2):

GFP before cor = GFP before − GFP bg and GFP after cor = GFP after − GFP bg(2)

The midline crossing index (MCI) is calculated using Equation (3):

MCI = GFP after cor/GFP before cor(3)

A decrease in the MCI value relative to the control may be interpreted as a reduction in the proportion of axons successfully crossing the midline.

To determine whether the axonal bundle has formed correctly, the ipsilateral corpus callosum trajectory index (ICTI) can be calculated. As a first step, the GFP intensity threshold must be set using the Threshold function (Image → Adjust → Threshold) and applied (Apply) to obtain a binary image of the GFP^+^ signal. Identical thresholds must be used throughout, and the resulting image must be visually verified against the original axonal tract. Next, within the ipsilateral hemisphere, an ROI for the intermediate zone (ROI IZ), encompassing the axonal tract, and, directly above it, an ROI for the cortical plate (ROI CP) must be defined (Figure 8D). The GFP^+^ signal areas in each ROI are then measured using the function Analyze → Measure, yielding the values Area IZ and Area CP. The ICTI is calculated using the Equation (4) where a decrease in ICTI is interpreted as a redistribution of axons from the IZ into the CP, which is characteristic of defects in axonal fasciculation:

ICTI = Area IZ/(Area IZ + Area CP)(4)

To standardise the results relative to control values, ICTI can be expressed as a percentage as shown in Equation (5):

ICTI percent = (ICTI exp sample/Mean (ICTI control)) × 100(5)

Thus, Fiji (ImageJ) enables standardized quantification of axonal projections. Manual tracing together with MCI and ICTI provides reproducible morphometric measures that are directly comparable across experimental groups. For valid comparisons, it is essential that control and experimental samples are matched in terms of the electroporated area and targeted brain region.

In a similar manner, Yan K. et al. measured the length of the axonal tract and its fasciculation, followed by calculation of the ICTI of the corpus callosum at E18.5, after IUE of EfnA4 and TrkB knockdown constructs at E13.5. The results of this study established that EphrinA reverse signalling directs the growth of the callosal tract within the ipsilateral hemisphere [213]. In another study, Chun-Lei Wang and colleagues analyzed the state of the corpus callosum at postnatal stages of development following overexpression of *Kir2.1* and *TeNT-LC* at E15.5. At stage P4, the authors quantitatively assessed the navigation of “pioneer” callosal axons by measuring the distance from the ML to three pioneer axons. It was shown that upon *Kir2.1* overexpression callosal axons lagged behind the pioneer axons when crossing the midline. In addition, at stages P7-P25 they performed a quantitative analysis of axons growing into the contralateral hemisphere within the somatosensory cortex. Quantification was carried out in layer IV of area S1, as well as in the S1/S2 border zone. In this case the region analyzed comprises three 500-µm-wide zones, in each of which the number of axons is counted. The results showed that overexpression of *Kir2.1* and *TeNT-LC* leads to an altered arborization pattern: increased branching was observed in superficial layer I, apparently accompanied by reduced branching in layers II–III [249].

In the work of Noda M. et al., MCI assessment established that knockdown of the circadian clock gene Per3 leads to a reduction in axon density in the contralateral hemisphere of the cerebral cortex [236]. An analogous approach based on measuring GFP intensity within defined regions of interest (ROIs) was employed by Ji L. et al., who showed that IUE of shRNA against INPP4B suppresses the medially directed outgrowth of callosal axons [253]. In the study by Sriwatsa et al., quantitative assessment of the fluorescence intensity area of axonal fibres and its subsequent normalisation to the area of the electroporated CP region demonstrated that the transcription factors Satb2 and Ctip2 regulate the choice of cortical projections through control of receptors of the Netrin1-DCC/Unc5C pathway. Satb2 represses *DCC* and Ctip2 represses *Unc5C,* thereby establishing a balance of attractive and repulsive signals and thus influencing the choice between callosal and subcortical projections [254]. These findings have been extended by Bessa et al., where they identified another target of Satb2—*Sema7a*, which controls callosal axons navigation [107]. 

In the study of neurons following IUE, the ex vivo approach based on electroporation and the subsequent preparation of organotypic cultures is no less noteworthy. In contrast to the analysis of fixed preparations, this approach makes it possible to study neurogenesis—and in particular axon growth and navigation—dynamically by means of live imaging. Such protocols are presented in more detail in the following articles: [255,256,257]. Several examples of the use of this approach are considered below.

In one study, Hand E. and colleagues employed live imaging to analyse the mechanisms of growth and laminar innervation of the cortex by the axonal projections of layer II/III neurons. To this end, IUE was performed in area S1 at stage E15.5; axonal growth was monitored using an EGFP-encoding plasmid, whereas collateralisation was visualized by labeling with multi-address genome-integrating color (MAGIC) markers cloned into the Brainbow vector system. Further analysis was carried out at postnatal stages in 400-µm organotypic slices immobilized in collagen gel and cultured under continuous perfusion with carbogen-saturated artificial cerebrospinal fluid. Prior to live imaging of axonal growth, the slices were allowed at least 2 h to recover. To assess cytoskeletal dynamics and filopodia formation, a plasmid encoding UtrCH probes, which bind F-actin, was additionally co-electroporated, thereby enabling visualisation of actin-dependent structures and their remodelling over time. The authors showed that the selective stabilization and branching of axonal collaterals within the target cortical layers are regulated predominantly by local microenvironmental cues and, according to their data, are independent of endogenous neuronal activity [258,259].

In a similar manner, Turchetto S. et al. visualized axonal transport of organelles in callosal axons. For this purpose, IUE was performed at stage E14.5 using plasmids expressing the selective markers Lamp1 and mito, which specifically label lysosomes and mitochondria, respectively. The choice of stage E14.5 allowed transfection of a restricted population of upper-layer cortical neurons, thereby enabling the visualisation of isolated fluorescent callosal axons and the analysis of organelle transport dynamics [256].

### 4.3. Quantitative Assessment of Neuronal Morphology

Quantitative analysis of neuronal morphology makes it possible to relate molecular and genetic alterations to measurable cellular phenotypes. In the course of postmitotic maturation, neurons form a branched dendritic tree and dendritic spines, which reflect the degree of their structural maturity and integration into neural networks [260]. In practice, the most commonly used approaches for assessing these processes are neurite reconstruction and morphometry (length, number of processes, branching points), as well as Sholl analysis. In particular, the number of branching points and the total length of both apical and basal dendrites are evaluated [236,261].

**Figure 9:**
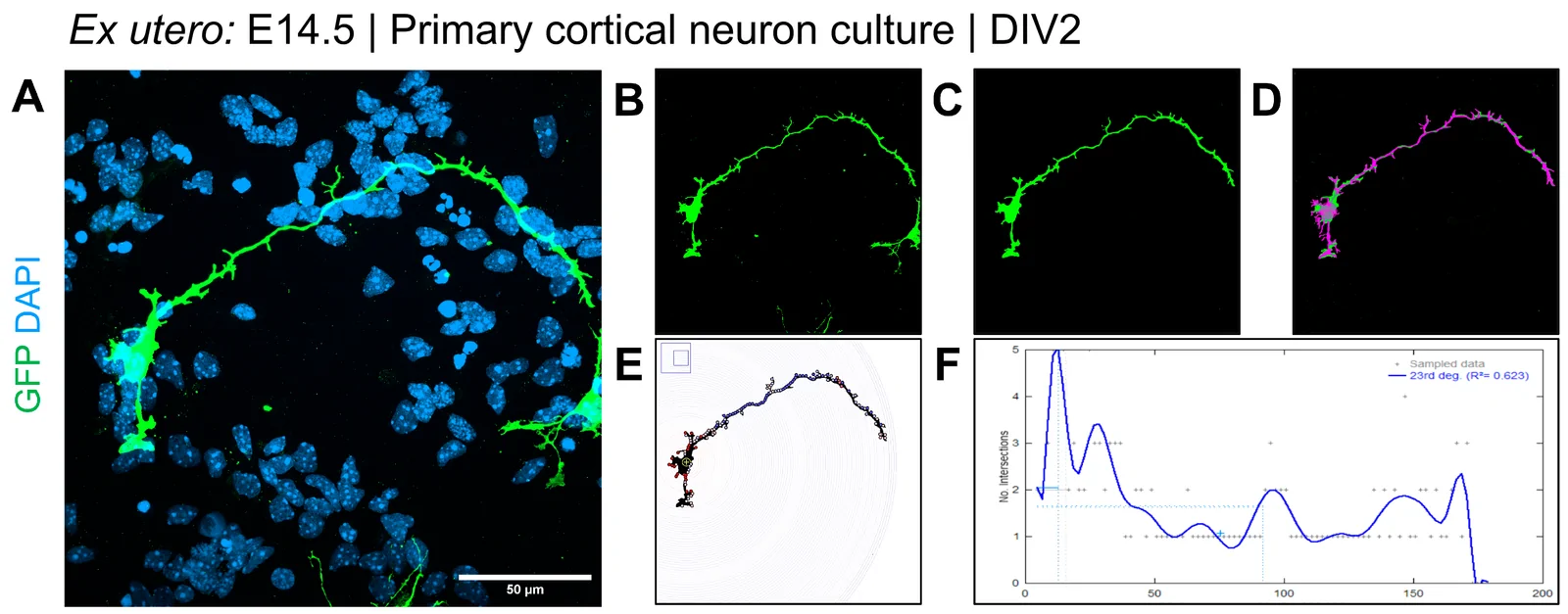
Analysis of neuronal morphology in Fiji using the SNT plugin: preparation of Z-stacks, neurite tracing and Sholl analysis. (**A**) Representative DIV2 cortical neuron after *ex utero* electroporation. (**B**)—Channel isolation for subsequent analysis. (**C**)—Image preprocessing and ROI restriction to a single neuron. (**D**)—Neurite reconstruction in SNT. (**E**)—Sholl analysis of the reconstructed neurite tree. (**F**)—Representative Sholl intersection profile. Green channel: detection of EGFP (Ex 488 nm, Em 510–530 nm). Blue channel: DAPI nuclear counterstain (Ex 358 nm, Em 461 nm). Created in Microsoft PowerPoint 2024 (Microsoft Corporation, Redmond, WA, USA). Adapted from previous work [262].

Let us consider the principal approaches to assessing these parameters in the Fiji application using the SNT (Simple Neurite Tracer) plugin based on confocal images obtained after *ex utero* electroporation of C57BL/6 mouse embryos at stage E14.5 using the plasmid vector pCAG-IG, followed by tissue dissociation, plating of primary neuronal cultures and analysis on the second day *in vitro* (DIV2). Z-stacks encompassing the entire neuron were acquired after GFP immunostaining and DAPI nuclear counterstaining (Figure 9A).

Following isolation of the GFP channel (Figure 9B) and image preprocessing to distinguish the selected neuron from background and neighboring cells (Figure 9C), SNT enables neurite reconstruction in 2D or 3D (Figure 9D). The resulting reconstructions allow quantification of total neurite length, individual branch lengths, and the numbers of primary neurites and branch points. Three-dimensional tracing additionally preserves the spatial geometry of processes.

Sholl analysis assesses neurite arbor complexity by counting intersections with concentric circles or spheres centered on the soma (Figure 9E). The resulting Sholl profile describes how the number of intersections varies with distance from the soma (Figure 9F).

As alternative software for reconstructing neurites from confocal Z-stack images, Neurolucida 2026.1.1 is used with subsequent analysis in Neurolucida Explorer [108,263]. Another useful tool is L-Measure v5.3, designed for the analysis of digital neuronal reconstructions with respect to various morphometric parameters, including total dendritic length, the number of primary dendrites and the number of branching points [108,264].

Thus, the use of these approaches ensures standardized, reproducible and quantitatively accurate analysis of neuronal morphological data, which is a prerequisite for comparisons between different experimental conditions.

In the study by Iyer et al., a comprehensive analysis of neurite morphology was performed both *in vitro* and *in vivo.* To this end, primary neurons were isolated from the cortex of mouse embryos at stage E14.5, transfected and analyzed at DIV5; in addition, IUE was performed at stage E15.5, with subsequent analysis of neurons at P14. The data obtained demonstrated that gain of function of the microRNAs *miR-193b* and *miR-365* leads to a significant reduction in the dendritic complexity of upper-layer cortical neurons (“*miR-193b-365* microcluster downstream of *Fezf2* coordinates neuron-subtype identity and dendritic morphology in cortical projection neurons”, iScience). In another study, Qiao H. et al. investigated the role of *USP7* in neuronal development. They carried out a series of transfections of primary hippocampal neuronal cultures at DIV4, 7 and 11, with subsequent morphological analysis 4 days after each transfection. Quantitative assessment of dendritic architecture was performed using Sholl analysis, as well as by counting the number of dendritic branches and measuring the total dendritic length. It was shown that overexpression of *USP7* leads to an increase in the number of dendritic branches and in total dendritic length, whereas *USP7* knockdown produces the opposite changes (“Role of the DUB enzyme USP7 in dendritic arborization, neuronal migration, and autistic-like behaviors in mice”, ScienceDirect). In the work of Liting Ji et al., it was shown that, following IUE, cortical neurons can be isolated and used for subsequent culture *in vitro.* The authors cultured GFP-positive cortical cells, fixed the cultures at DIV2 and performed immunohistochemical staining for GFP, Tau-1 and MAP2, after which they calculated the proportion of GFP^+^ cells without an axon, with a single axon or with multiple axons. At DIV3, morphometric analysis was carried out, including measurement of primary axon length in ImageJ using the NeuronJ plugin, as well as assessment of branching and neurite number using NeurphologyJ. The results of these experiments showed that depletion of the phosphatase INPP4B under study significantly suppressed axon formation in GFP-positive neurons, increasing the proportion of cells lacking axons, while having no effect on axon length, degree of branching or neurite [253]. Another study employing similar approaches was that of Kimura T. and Murakami F., devoted to the influence of mitochondria on the formation of the dendritic tree. Using IUE, they enhanced *Mfn1* expression, which resulted in increased branching of the proximal portion of dendrites [263].

Noteworthy is the use of a custom MATLAB 2019a (The MathWorks, Inc., Natick, MA, USA) script for the quantitative analysis of axon terminals. As a first step, confocal images are imported into MATLAB using the Bio-Formats library, after which they are pre-processed and the signal is segmented. The user then manually traces axons in the channel of the selected fluorescent protein, placing reference points along the process. The program subsequently identifies axon terminals automatically on the basis of the presynaptic marker synaptophysin-EGFP, matches them to the axon trajectory, measures the dimensions of the identified structures and calculates the length of each analyzed segment. The results are exported into a summary table, which makes it possible to calculate the linear density of terminals as the number of structures per unit axon length [265].

Further information on other programs for tracing and quantitative assessment of neuronal processes can be found in the following works: [266,267].

Following quantitative assessment of the dendritic tree, the next level of analysis is the study of dendritic spines, which reflect the degree of synaptic maturity of the neuron and the features of excitatory synapse formation. For the analysis of dendritic spines, it is recommended that confocal imaging of individual dendritic segments be performed with a Z-stack step of approximately 0.2–0.3 µm using a 63× objective and 2× digital zoom, which provides spatial resolution sufficient for spine morphometry. Subsequent analysis may be carried out in either automated or semi-automated/manual format. In particular, the commercial software IMARIS 8.0 (Oxford Instruments/Bitplane, Zurich, Switzerland) with the Filament Tracer module allows automatic assessment of spine density, spine length and head diameter, and classification of spines by morphological type (short, mushroom, long thin, filopodia) [114,268].

In a number of studies, the linear density of labeled puncta is used to assess labeled structures per unit dendritic length. In this case, the ratio of the number of punctate inclusions to the length of the corresponding dendritic segment is calculated. Dendritic spine morphology is often analyzed on 2D images obtained by maximum intensity projection of 3D stacks in Fiji, with subsequent manual reconstruction of clearly discernible spines in the SynPAnal program [269]. For example, an ROI 700 nm in diameter is placed around the spine head and, following thresholding of the signal, the proportion of spines containing PSD-95-GFP puncta is determined. GFP-gephyrin structures were detected after image thresholding using the Analyze Particles tool in Fiji [108]. Using such a design, it was shown that *Dcx* knockdown is accompanied by an increase in spine head and neck diameter, indicating the involvement of Dcx in synaptogenesis.

In addition, a simplified formalised method for spine assessment on 2D images without specialised software has been described. In this approach, the major (D major) and minor (D minor) axes of the spine head are measured, after which its surface area is calculated using the Equation (6):

¼ × π × D major × D minor(6)

Spines with a calculated head area of approximately ≥0.4 µm^2^ are classified as mushroom spines [270].

Thus, the approaches presented constitute a unified, standardized toolkit for the interpretation of phenotypes arising from genetic modulation *in vivo* and *in vitro.* The combined analysis of axonal tract length and of the MCI and ICTI indices, together with quantitative assessment of the dendritic tree and dendritic spines, broadens and strengthens the methodological basis for interpreting the effects observed after IUE (Table 2). The application of uniform image preparation procedures, strictly defined regions of interest (ROIs), and reproducible metrics ensures direct comparability of results between experimental groups and minimises the contribution of technical variability, which is particularly important when detecting subtle morphological changes.

Thus, the application of IUE in combination with post-processing of confocal images has substantially expanded the possibilities for studying the morphological features of neurons, ranging from the formation of axonal and dendritic processes to neuronal networks and commissures.

## 5. Conclusions

*In utero* electroporation is a method that allows brain development to be studied at both embryonic and postnatal stages, and it has become one of the key tools available to researchers. This is due to its relatively simple technical implementation, which provides spatiotemporal control of genetic manipulation and cellular specificity through the choice of an optimal promoter, embryonic stages and the experimental design itself. Combining IUE with thymidine analogues (BrdU, EdU, CldU, IdU) makes it possible to quantify cell cycle kinetics, the birthdates of transfected cells and their exit from the proliferative pool. In conjunction with staining for cell cycle markers (Ki-67, PH3) and specific laminar markers, it is possible to characterize in detail the impact of genetic modifications on the balance between the proliferation and differentiation of neural progenitor cells, on laminar distribution, and on neuronal migration and specification. Additional image post-processing (quantitative assessment of the fasciculation and density of axonal projections, neurite tracing, Sholl analysis) using the software described makes it possible to link molecular perturbations to specific alterations in the formation of neuronal networks both *in vitro* and *in vivo.*

At the same time, IUE has a number of limitations. The method requires precise and professionally executed surgical intervention. Improper technique may give rise to artifacts and false-positive results, which necessitates mandatory verification using immunohistochemical markers, as well as analysis of a representative sample.

Despite these limitations, IUE holds considerable promise for further development and application in studies of brain development. Combining *in utero* electroporation with organotypic cultures, *in vivo* and ex vivo time-lapse microscopy [257], single-cell genomic and transcriptomic profiling [272], and Ca^2+^ imaging [273] opens up the possibility of directly relating molecular, morphological and functional phenotypes in the developing brain. What is particularly promising is the use of IUE for modelling somatic mosaic mutations, which are increasingly regarded as a cause of congenital malformations and neuropsychiatric disorders [274].

The application of IUE is particularly promising for functional studies of genes associated with impaired embryonic development and for modeling the consequences of altered expression of such genes in the developing brain [275]. For example, suppression of *ANK2* expression by IUE in the developing mouse cortex disrupted the differentiation of neural stem cells and neuronal migration and altered the expression of genes involved in nervous system development. These results point to a possible role of *ANK2* deficiency in the pathogenesis of autism spectrum disorders. In another study, knockdown of the schizophrenia risk gene *DGCR2* by IUE led to impaired laminar distribution of pyramidal neurons and altered dendritic tree structure and dendritic spine maturation in the medial prefrontal cortex. Electrophysiological analysis further revealed a decrease in the frequency and amplitude of excitatory and inhibitory postsynaptic currents, indicative of impaired functional maturation of local neural circuits [276].

Thus, *in utero* electroporation (IUE) represents a versatile tool for studying the development of the cerebral cortex, enabling investigation of cell cycle regulation, neuronal differentiation and migration, the formation of cortical layers, and the development of neural networks. Combining this technique with the analysis of specific molecular markers and functional approaches further expands the possibilities for elucidating the molecular mechanisms underlying corticogenesis and for modeling neurodevelopmental pathologies. Despite the need for rigorous control of surgical procedures and careful exclusion of experimental artifacts, the continued integration of IUE with modern imaging and molecular analysis techniques offers promising opportunities for establishing causal relationships between genetic alterations, disruptions in cellular development, and the emergence of pathological phenotypes in the developing brain.

## Figures and Tables

**Table 1:** Comparative overview of *in vivo* approaches to studying brain development.

Feature	*In Utero* Electroporation (IUE)	Transgenic Models (Cre/Lox, etc.)	Viral Delivery Systems (AAV, Lentivirus)	Ultrasound-Guided *In Utero* Transplantation (IUT) of Labeled Cells IUE
Targeting Specificity	The targeted region is defined by electrode placement, and the identity of the labeled population depends on the embryonic stage. Promoters and genetic switches further confine expression [33,34].	Promoter-driven systems confer cell-type specificity but carry a risk of off-target expression [35].	High cell-type specificity via capsid-promoter synergy, limited by interstitial vector diffusion [36,37].	High macro-spatial targeting via real-time ultrasound guidance, limited by post-transplantation cell migration and developmental plasticity restrictions [38,39].
Efficiency	While the resulting population is typically mosaic and coverage depends on the target region, developmental stage, and construct, IUE demonstrates a procedure success rate of 81.3–87.5% with high fetal viability, yielding localized transfection and a mosaic expression profile [33,34].	Transgene presence does not guarantee complete recombination (dependent on the driver, locus, and induction); maximum efficiency (up to 100%) requires a loxP inter-site distance of <4 kb [40].	High transduction rates within the viral diffusion radius depend on the vector, dose, tissue, and delivery route [41,42].	Depends on cell survival and successful integration of donor tissue [39].
Duration of Gene Expression	Episomal (non-integrating) plasmids are diluted out with each cell division, whereas in postmitotic cells, expression may remain prolonged [43].	Cre-mediated recombination induces a stable genomic alteration that is maintained in daughter cells. Recombination can occur constitutively or be temporally induced by tamoxifen in CreERT/CreERT2 systems, enabling controlled gene modification [44].	AAV vectors provide prolonged transgene expression in post-mitotic cells, with vector genomes persisting predominantly as episomal circular and concatemeric forms. In contrast, integration-competent lentiviral vectors support long-term transgene expression through stable genomic integration [45,46,47].	Expression longevity is contingent upon the biological properties of the donor cells [48].
Technical Complexity	The integration of embryonic microsurgery, specialized electroporator utilization, and reproducible electrode placement dictates stringent requirements for operator technical proficiency and manual dexterity [34].	Time-consuming generation and validation of transgenic lines, followed by on-going breeding and colony maintenance. Cre/loxP models may require multiple rounds of crossbreeding to obtain the desired genotype, routine genotyping, and periodic validation of transgene expression and recombination activity [49,50].	AAV- and lentiviral delivery require vector production, titration, and specialized injection equipment, as well as precise anatomical targeting and extensive optimization of dose, serotype, and developmental stage [51,52].	Technically demanding, requiring donor cell preparation and labeling, specialized high-frequency ultrasound guidance for precise embryonic targeting and microinjection, followed by assessment of donor cell engraftment, distribution, and graft function [53,54].
Main Experimental Limitations	Mosaic transgene expression, variable localization of transfected cells, and the risk of embryonic injury are key limitations of IUE. In addition, plasmid dilution during cell division may reduce transgene expression over time [55].	Cre-mediated recombination may be incomplete, variable across target genes, or occur in unintended cell types. Inducible systems introduce additional confounding factors, including tamoxifen-related effects and unintended recombination, complicating the interpretation of experimental outcomes [56,57,58].	Viral delivery systems are limited by the small packaging capacity of AAV vectors (~4.7 kb), capsid- and context-dependent tropism, and potential immune and inflammatory responses that may reduce transduction efficiency or damage transduced cells. Integration-competent lentiviral vectors carry an additional risk of insertional mutagenesis due to genomic integration [59,60,61,62].	Donor cell fate and engraftment depend on the host brain microenvironment. Engraftment does not guarantee functional integration into neural circuits. Moreover, IUT does not directly enable genetic manipulation of endogenous host cells [38,63].

**Table 2:** Applications of software for the analysis of neuronal processes.

Program, Plugin	Functions Used	Developmental Stage	Examples
ImageJ/Fiji	measurement of fluorescence signal intensity to assess the density of callosal projections	P2	[236,251,253,271]
Neurolucida	axon tracing, delineation of the boundaries of the corpus callosum	P8	[250]
ImageJ, NeuronJ plugin	determination of axon length	DIV3	[253]
ImageJ, NeurphologyJ plugin	number of endpoints and neurites	DIV3	[253]
ImageJ, Simple Neurite Tracer plugin	dendrite tracing	P7	[268]
ImageJ, Sholl Analysis plugin	Sholl analysis	P2–7	[261]
Neurolucida with subsequent analysis in Neurolucida Explorer	reconstruction of dendrites from confocal Z-stack images	P14–15 (rats)	[108,263]
L-Measure	analysis of digital neuronal reconstructions	P4–5	[108,264]
IMARIS with the Filament Tracer module	automatic assessment of spine density, length and head diameter, and classification of spines into types	stage not specified	[114]
Fiji, Cell Counter	cell counting	stage not specified	[108]
Fiji, SynPAnal	delineation of dendritic regions using a fluorescence threshold to determine the proportion of spines containing puncta	stage not specified	[269]
Fiji, Analyze Particles	detection of puncta after thresholding	stage not specified	[108]

## Data Availability

No new data were created or analyzed in this study. Data sharing is not applicable to this article.

## Abbreviations

The following abbreviations are used in this manuscript:

4-OHT	4-hydroxytamoxifen
AMPK	AMP-activated protein kinase
AAVs	adeno-associated viruses
BrdU	5-Bromo-2′-deoxyuridine
CAG	CMV early enhancer/chicken β-actin
CEP85L	Centrosomal protein of 85 kDa-like
CFTR	cystic fibrosis transmembrane conductance regulator
CldU	5-Chloro-2′-deoxyuridine
CMV	Cytomegalovirus
CNS	Central nervous system
CP	Cortical plate
CreERT2	Tamoxifen-inducible Cre recombinase (second generation)
CRISPR	Clustered regularly interspaced short palindromic repeats
CT	callosal tract
Ctip2	COUP-TF-interacting protein 2
DAPI	4′,6-Diamidino-2-Phenylindole
DCC	Deleted in colorectal cancer
Dcx	Doublecortin
DIV	Day *in vitro*
DNA	Deoxyribonucleic acid
dsDNA	double-stranded DNA
DS-gRNA	donor-specific gRNA
dUTP	Deoxyuridine triphosphate
Easi-CRISPR	Efficient addions with ssDNA inserts-CRISPR
EBFP	Enhanced Blue Fluorescent Protein 2
EdU	5-Ethynyl-2′-deoxyuridine
E	Embryonic day
EfnA4	ephrin-A4
EGFP	Enhanced Green Fluorescent Protein
ESC	Embryonic stem cell
Fezf2	FEZ family zinc finger 2
Gal-T2	Galactosyltransferase 2
GFAP	Glial fibrillary acidic protein
GFP	Green fluorescent protein
Gli3	GLI family zinc finger 3
GLAST	Glutamate–aspartate transporter
gRNA	guide RNA
HDR	homology-directed repair
HITI	homology-independent targeted integration
HiUGE	Homology-Independent Universal Genome Engineering
ICTI	Ipsilateral corpus callosum trajectory index
IdU	5-Iodo-2′-deoxyuridine
i-GONAD	Improved Genome-editing via Oviductal Nucleic Acids Delivery
IGF-1R	Insulin-like growth factor 1 receptor
INPP4B	Inositol polyphosphate-4-phosphatase type II B
iPSC	Induced pluripotent stem cell
IRES	internal ribosome entry site
IUE	*In utero* electroporation
IUT	*in utero* transplantation
IZ	Intermediate zone
Ki67	Kiel 67 antigen
Kir2.1	Inward rectifier potassium channel 2.1
LAMP1	Lysosome-associated membrane protein 1
LM (TK−) cell	L-M derivative (Thymidine kinase-deficient) mouse fibroblast cell line
LVs	lentiviruses
MAGIC	Multi-address genome-integrating color
MAP2	Microtubule-associated protein 2
MCI	Midline crossing index
Mfn1	Mitofusin-1
ML	Midline
mKO1	monomeric Kusabira-Orange 1
MPPH	megalencephaly, postaxial polydactyly, polymicrogyria, and hydrocephalus
mRNA	Messenger ribonucleic acid
MTSS2	MTSS I-BAR domain containing 2
MZ	Marginal zone
NHEJ	non-homologous end joining
NSC	Neural stem cell
ORANGE	Open Resource for the Application of Neuronal Genome Editing
ORF	Open reading frame
P	Postnatal day
PACT	Protein activator of the interferon-induced protein kinase
PBS	Phosphate-buffered saline
Per3	Period circadian regulator 3
pCIG	plasmid CAG-IRES-GFP
PH3	phospho-Histone H3
PVH	Periventricular heterotopia
RHEB	Ras homolog enriched in brain
RISC	RNA-induced silencing complex
RFP	Red fluorescent protein
RGC	Radial glial cell
RNA	Ribonucleic acid
ROI	Region of interest
RP58	Repressor protein 58
RT	Recombinase target site
Satb2	Special AT-rich sequence-binding protein 2
SeCre	self-excising Cre
Sema7a	Semaphorin 7A
shRNA	small hairpin RNA
SLENDR	single-cell labeling of endogenous proteins by CRISPR-Cas9-mediated homology-directed repair
SNT	Simple Neurite Tracer
ssDNA	single-stranded DNA
ssODN	single-stranded oligodeoxynucleotide
SSR	Site-specific recombinase
SVZ	Subventricular zone
Syn1	Synapsin I
Tau-1	tubulin associated unit-1
TBR1	T-box brain transcription factor 1
Tbr2	T-box brain protein 2
TeNT-LC	Tetanus neurotoxin light chain
Thy1	Thymocyte antigen 1
tTA	tetracycline-controlled transactivator
TRE	Tetracycline response element
TrkB	Tropomyosin receptor kinase B
Tuba1a	Tubulin Alpha 1a
Tuj1	B-III tubulin
TUNEL	Terminal deoxynucleotidyl transferase dUTP nick end labeling
Unc5C	Unc-5 netrin receptor C
USP7	Ubiquitin-specific protease 7
UtrCH	Utrophin calponin homology domain
VZ	Ventricular zone
XFP	X fluorescent protein
YFP	Yellow fluorescent protein
